# Reconstruction of the Origin of a Neo-Y Sex Chromosome and Its Evolution in the Spotted Knifejaw, *Oplegnathus punctatus*

**DOI:** 10.1093/molbev/msab056

**Published:** 2021-03-09

**Authors:** Ming Li, Rui Zhang, Guangyi Fan, Wenteng Xu, Qian Zhou, Lei Wang, Wensheng Li, Zunfang Pang, Mengjun Yu, Qun Liu, Xin Liu, Manfred Schartl, Songlin Chen

**Affiliations:** 1 Yellow Sea Fisheries Research Institute, CAFS; Laboratory for Marine Fisheries Science and Food Production Processes, Pilot National Laboratory for Marine Science and Technology (Qingdao), Qingdao, China; 2 College of Fisheries and Life Science, Shanghai Ocean University, Shanghai, China; 3 Key Laboratory of Sustainable Development of Marine Fisheries, Ministry of Agriculture, Qingdao, China; 4 BGI-Qingdao, BGI-Shenzhen, Qingdao, China; 5 Laizhou Mingbo Aquatic Product Co. Ltd., Laizhou, Shandong, China; 6 Entwicklungsbiochemie, University of Würzburg, Biozentrum, Würzburg, Germany; 7 Xiphophorus Genetic Stock Center, Texas State University, San Marcos, TX, USA

**Keywords:** neo-Y, evolution, spotted knifejaw, genome

## Abstract

Sex chromosomes are a peculiar constituent of the genome because the evolutionary forces that fix the primary sex-determining gene cause genic degeneration and accumulation of junk DNA in the heterogametic partner. One of the most spectacular phenomena in sex chromosome evolution is the occurrence of neo-Y chromosomes, which lead to X1X2Y sex-determining systems. Such neo-sex chromosomes are critical for understanding the processes of sex chromosome evolution because they rejuvenate their total gene content. We assembled the male and female genomes at the chromosome level of the spotted knifejaw (*Oplegnathus punctatus*), which has a cytogenetically recognized neo-Y chromosome. The full assembly and annotation of all three sex chromosomes allowed us to reconstruct their evolutionary history. Contrary to other neo-Y chromosomes, the fusion to X2 is quite ancient, estimated at 48 Ma. Despite its old age and being even older in the X1 homologous region which carries a huge inversion that occurred as early as 55–48 Ma, genetic degeneration of the neo-Y appears to be only moderate. Transcriptomic analysis showed that sex chromosomes harbor 87 genes, which may serve important functions in the testis. The accumulation of such male-beneficial genes, a large inversion on the X1 homologous region and fusion to X2 appear to be the main drivers of neo-Y evolution in the spotted knifejaw. The availability of high-quality assemblies of the neo-Y and both X chromosomes make this fish an ideal model for a better understanding of the variability of sex determination mechanisms and of sex chromosome evolution.

## Introduction

Sex chromosomes are the most peculiar components of the genome, and they appear in a great variety of forms throughout the plant and animal kingdom. As carriers of the genetic sex-determining loci, they follow their fate: mutation, degeneration, gene loss, or turnover (Abbott et al. 2017). Sex chromosomes arise and disappear repeatedly and independently, in some cases, even in closely related species. The origin and evolution of sex chromosomes are central topics in evolutionary genetics because of their association with what Maynard Smith called the “queen of problems,” the evolution of sex (Maynard Smith 1978). In fulfilling Fisher’s postulate for a 1:1 sex ratio (Fisher 1930), male and female heterogamety are the dominant systems. It is commonly believed that all sex chromosomes follow the same trajectory of evolution. At first, to keep their chromosomal identity, recombination ceases around the emerging sex determination locus on the proto-sex chromosomes and finally becomes absent over almost the entire W and Y chromosomes. Linkage disequilibrium (LD) with a gene that is beneficial for one sex and/or detrimental to the opposite sex is predicted to facilitate this first step (Kirkpatrick 2017). Over time, reduced recombination causes the Y and W chromosomes to diverge from their gametologs and become heteromorphic in size. Additionally, they often have specialized gene content that often requires dosage compensation (Charlesworth et al. 2005; Kejnovsky et al. 2009). The Y or W chromosomes of many species (including all genetic model species such as mouse, chicken, *Drosophila*, etc.) have lost most of their genes over tens of millions or over 100 My. During their long evolutionary history, their DNA content became highly repetitive. The old age and the gene-poor, repeat rich DNA content of the classical Y and W chromosomes considerably impedes studies on their evolutionary history and the mechanisms that drove their development. Younger sex chromosomes therefore provide good models to study sex chromosome evolution. In particular, teleost fish are outstanding objects to study this topic because they present a broad range of sex chromosome systems (Volff 2005; Piferrer et al. 2012). All fish species studied so far have much younger sex chromosomes compared with mammals and birds, making it possible to analyze the early stage of sex chromosome evolution and differentiation at the molecular level (Charlesworth et al. 2005; Bachtrog 2006; Cioffi and Bertollo 2010). Heteromorphic sex chromosomes have been found only in about 10% of karyotyped fish species, whereas most species have morphologically undifferentiated, homomorphic sex chromosomes.

Turnover of sex-determining mechanisms and sex chromosomes is a frequent phenomenon in various groups of plants and animals (Herpin and Schartl 2015; Schartl et al. 2016). Multiple mechanisms were suggested based on cytogenetic evidence, including the transposition of an existing male-determination locus to an autosome (Woram et al. 2003), the emergence of a new male-determination locus on an autosome (Kondo et al. 2006; Tanaka et al. 2007), and fusions between an autosome and an existing Y chromosome (Kitano et al. 2009; Ross et al. 2009). However, for a deeper understanding of sex chromosome structure and evolution, knowledge of their entire sequence is required. Despite the great opportunities that new sequencing technologies offer in fish, they have only rarely and reluctantly been applied to the sex chromosomes, as they are the most difficult part of the genomes to study. Most fish genome projects have purposely avoided the heterogametic sex.

Neo-Y chromosomes present a peculiar situation where an already differentiated Y chromosome fuses with an autosome and forces the former autosome to evolve the stereotypical properties of ancestral sex chromosomes. The homologous partner becomes a new X chromosome (now termed X2) and is now subject to the special forces that drove the evolution of the older X (X1). Intriguingly, this leads to a situation where the male karyotype has one chromosome less than the female karyotype. Neo-Y chromosomes have been intensively studied in different lineages (Kitano et al. 2009; Cioffi and Bertollo 2010; Pala et al. 2012), but so far, only *Drosophila miranda* and *Oplegnathus fasciatus* have been sequenced (Mahajan et al. 2018; Xiao et al. 2020). We report here the long-read technology-based chromosome assemblies of a female and male spotted knifejaw (*O. punctatus*) including their three complete sex chromosomes. This allowed us to reconstruct the evolutionary history of the neo-Y formation and both X chromosomes and their peculiar gene content.

## Results

Spotted knifejaw fish are an emerging aquaculture species of high economic value in East Asia. Previous conventional cytogenetic analysis showed a female karyotype of 2*n* = 48 chromosomes (2m + 46a), whereas the male has 2*n* = 47 chromosomes (3m + 44a; Li et al. 2016), which indicated a multiple sex chromosome system with X1X1X2X2 chromosomes in females and X1X2Y chromosomes in males. The Y chromosome is metacentric and easily recognized by its large size (Li et al. 2016). In contrast, the X1 and X2 chromosomes are acrocentric, similar to most autosomes, which makes them difficult to identify and to confirm and study the sex chromosome system.

### Sequencing and Genome Assembly

To reconstruct the origin of the neo-Y chromosome and the evolutionary processes that shaped its history, we sequenced and assembled the genomes of a female and male spotted knifejaw. Using 123.65 Gb (161-fold genome coverage) 10X Genomics^TM^ linked reads and 121 Gb Hi-C reads, 764 Mb of the estimated 768 Mb female genome was assembled with a scaffold N50 of 29.92 Mb (supplementary table S1, Supplementary Material online). A total of 718 Mb of the genome sequence (∼92% of the assembly) is contained in 24 large scaffolds matching the chromosome number of the female karyotype (supplementary fig. S1*a*, Supplementary Material online). The lengths of the female chromosomes correlate well with their physical length described in a previous karyotype study (Li et al. 2016) (*R*^2^ = 0.97, supplementary fig. S1*b*, Supplementary Material online). Because of the considerable challenges that degenerated sex chromosomes pose for assembly, we added another 73.2 Gb (96-fold) Nanopore and 130.31 Gb (170-fold) PacBio long reads to the first-round sequencing of the 153.56 Gb (201-fold) linked reads and 148.75 Gb Hi-C reads of the male genome. This resulted in an assembly of 831 Mb with scaffold N50 of 29.98 Mb, which is slightly larger than the 763 Mb estimate from *k*-mer analysis (Li et al. 2010).

**Fig. 1.**
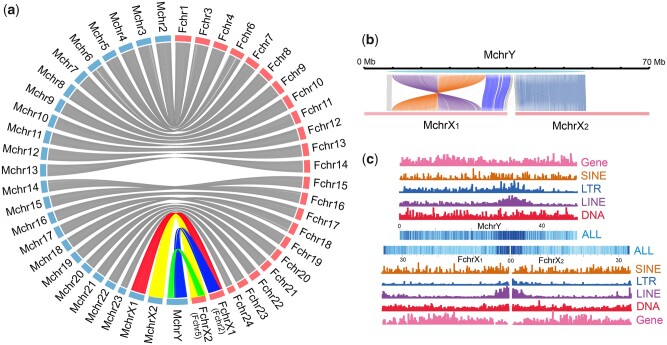
The male and female genome assembly of the spotted knifejaw. (*a*) Synteny relationship of male (left side, blue) and female (right side, pink) chromosomes. Lines linking two chromosomes indicate the location of homologs, with gray lines connecting autosomes and multicolored lines representing the relationship between sex chromosomes of male and female (red for X1, yellow for X2, blue for gametologs of X1 and neo-Y, green for gametologs of X2 and neo-Y). (*b*) One-to-two synteny relationships of the two arms of neo-Y with X1/X2. MchrY designates neo-Y (light blue), whereas FchrX1 and FchrX2 are the X chromosomes (pink). Synteny relationships of the inversion region are shown in purple and brown, whereas those of the PARs are in light gray, the centromere region is in dark gray, and the other regions are in blue. (*c*) Repetitive sequence analysis. The upper depicts MchrY (Y), the lower depicts FchrX1 (X1) and FchrX2 (X2). Repetitive sequence types are indicated on the right. Note that the enrichment of LINE and LTR at the fusion point on neo-Y and regions on the corresponding centromeric regions of X1 and X2.

The quality of the female and male genome assemblies was assessed by the coverage of the Actinopterygii core genes of the BUSCO pipeline (Actinopterygii_odb9), yielding 96.4% and 96.8%, respectively, completely covered (supplementary table S2 and supplementary fig. S2, Supplementary Material online). Importantly, the high divergence region of the Y chromosome is contained in one continuous scaffold sequence with a length of ∼48.5 Mb (supplementary fig. S3, Supplementary Material online). The chromosome assembly matches the 25 chromosomes of the male karyotype, consistent with 22 autosomes, two X chromosomes and one Y chromosome (fig. 1*a*). We then annotated 22,970 and 25,234 genes for the female and male genomes, respectively.

Although 22 chromosomes covering more than 96% (supplementary table S3, Supplementary Material online) of the male and female genome show a full correspondence in the synteny analysis, there is a shared synteny relationship of two chromosomes (Fchr2 and Fchr5, now termed FchrX1 and FchrX2) of the female to the two arms of one large chromosome in the male (fig. 1*a*). Additionally, these two chromosomes in the female show a good synteny relationship with two other chromosomes in the male (MchrX1 and MchrX2) separately. This clearly identifies the Y, X1, and X2 chromosomes of the spotted knifejaw and shows that the Y chromosome of the spotted knifejaw is a neo-Y chromosome that arose by fusion of an ancient Y (the gametolog of X1) with the new X2’s precursor.

The predicted fusion point in the neo-Y chromosome is highly enriched for repetitive sequences, which may create assembly errors (supplementary fig. S4, Supplementary Material online**)**. However, correctness was confirmed by the fact that approximately half of the 10X Genomics (∼30X read depth of total 56X), Nanopore (∼20X of 40X), and PacBio (∼20X of 40X) reads unambiguously bridged the fusion point, and the remaining half (derived from X1 and X2) were discontinuously aligned into sequences from the two arms flanking the fusion point (supplementary fig. S5, Supplementary Material online). The abundance of repetitive sequences in the fusion region of the neo-Y chromosome, which corresponds to the centromeric regions at the tips of the acrocentric X1 and X2 (fig. 1*c*), suggests that a Robertsonian translocation generated the neo-Y of the spotted knifejaw. The neo-Y chromosome has twice as many pseudogenes (8.4%) as X1 (3.9%), X2 (3.8%), and the autosomes (3.3%) (supplementary fig. S6*a* and supplementary table S4, Supplementary Material online). On the neo-Y, there are more pseudogenes in the inversion region (homologous to X1) than that in the region homologous to X2 (supplementary fig. S6*b*, Supplementary Material online). Repeat element annotation (supplementary tables S5 and S6, Supplementary Material online) indicates that LINE and long terminal repeat (LTR) elements appear to have accumulated more recently, specifically on the neo-Y chromosome (supplementary fig. S7, Supplementary Material online).

### Molecular Differentiation of the Neo-Y and X Chromosomes

**Fig. 2.**
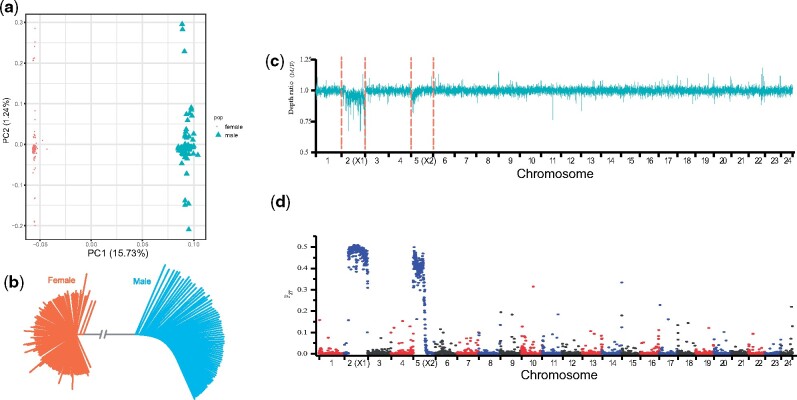
Genome-wide distribution of SNPs from 73 male and 124 female individuals. (*a*) Principal component analysis of 197 individuals using SNPs. (*b*) Phylogenetic tree showing relationships of male (blue) and female (orange) SNPs. (*c*) Average sequencing depth ratio between females and males. The read depth distribution in both female and male was similar for all autosomes, whereas it was decreased for most of X1 and the proximal region of X2. (*d*) Genome-wide scan of fixation index (*F*_ST_) matching the result from the read depth distribution.

To characterize the degree of molecular differentiation of sex chromosomes and to identify sex-specific genomic regions possibly involved in sex chromosome evolution, we sequenced 73 male and 124 female individuals with an average depth of ∼11-fold (supplementary table S7, Supplementary Material online). Using the female genome as a reference, we detected a total of 4.27 million single nucleotide polymorphisms (SNPs). Principal component analysis (fig. 2*a*) and an SNP tree (fig. 2*b*) showed that male and female genomes cluster into two distinct groups, indicating sex-specific differentiated genomic regions. The average sequencing depth ratio in both females and males was similar for all autosomes, whereas for the X1HDR (high divergence region corresponding to X1) and X2HDR, it was lower than that for autosomes but higher than 0.5 (fig. 2*c*). A genome-wide association study (GWAS) using the compressed mixed linear model (cMLM) implemented in GAPIT showed that the regions of 29.3 Mb of X1 (from 0 to 29.30 Mb, X1HDR) and 17.58 Mb of X2 (from the centromere to 17.58 Mb, X2HDR) were associated with sex (supplementary fig. S8, Supplementary Material online). The genome-wide fixation index (*F*_ST_) (Weir and Cockerham 1984) between males and females also showed substantially higher differentiation in almost the same two regions (fig. 2*d* and supplementary fig. S8, Supplementary Material online). Finally, the LD values on X1 and X2 were substantially higher than those on the autosomes (supplementary fig. S9, Supplementary Material online).

SNP density mapping revealed that all autosomes show a similar low SNP density of ∼0.3% between males and females. However, X1HDR and X2HDR present a much higher SNP density in male individuals and substantially higher heterozygosity (supplementary fig. S10, Supplementary Material online). Approximately 23.9% of SNPs in X1HDR and X2HDR exhibit male-specific heterozygosity when mapped to the neo-Y haplotype. The average sequencing depth ratio between females and males for X1HDR and X2HDR was lower than that for autosomes but higher than 0.5 (fig. 2*c*), which indicates a certain degree of sequence similarity of the neo-Y chromosome with the ancestral X1 chromosome and an even higher similarity with the X2 chromosome. The results are in agreement with a mechanism in which an ancestral Y chromosome, which already had diverged considerably from the X1 chromosome, fused with an autosome (the counterpart of the proto-X2). This must have happened later during evolution and did not occur concomitantly with the origin of the Y chromosome.

### Origin and Evolution of the Neo-Y

Alignment of the neo-Y to X1 uncovered a large inversion spanning 23.5 Mb on the X1-syntenic arm. Both breakpoints of the inversion were fully supported by three types of sequencing reads (10X Genomics, PacBio, and Nanopore, supplementary fig. S5, Supplementary Material online). The inversion on the neo-Y contains 992 genes. We performed Gene Ontology (GO) classification of these genes and found that some of them were classified in GO terms chromosome and cell differentiation, such as centromere protein P, heterochromatin protein 1-binding protein 3, and synaptonemal complex protein 1 (supplementary fig. S11, Supplementary Material online). Based on the gene set, in the inverted region, 89 genes from the corresponding region of X1 are absent from the Y chromosome, whereas 139 genes have no homolog on the X1-syntenic arm and may be highly divergent XY (HD-XY) genes (supplementary table S8, Supplementary Material online). Through LiftOver and homology-based annotation, 24 of the 139 genes on the Y were found to have no homologous sequences on the X1-syntenic arm and may be named as Y^+^X^−^-genes. Whether these genes play male-specific roles remains unclear. Only one gene of the HD-XY genes is annotated by homology to known functional proteins as a ubiquitin protein ligase, whereas others show similarity to Transposable Elements (TEs) or copies of autosomal genes. Such accumulation of “junk” DNA is a hallmark of nonrecombining regions of sex chromosomes.

**Fig. 3.**
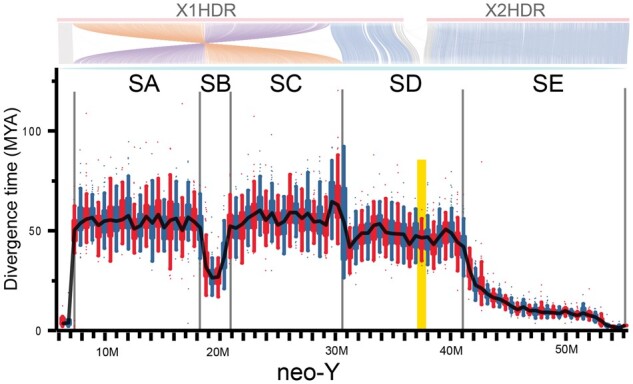
Divergence times along the neo-Y in a sliding window of 100 kb. SA: section A, SB: section B, SC: section C, SD: section D, SE: section E. Yellow bar: fusion region. SA, SB, and SC correspond to the inversion region. The region to the left of the yellow bar shows divergence times between X1 and Y, whereas the region to the right shows divergence times between X2 and Y. The synteny relationships between neo-Y and Xs (X1HDR and X2HDR) are shown in the upper part as described in the legend of figure 1. Note: The mutation rate (8.18 × 10^−10^ per site per year) of the spotted knifejaw was estimated through the comparative genomics analysis using ten other fish species. The genetic distance was calculated using collinearity blocks between the X1/X2 and the neo-Y within a 100 kb sliding window. The divergence time of each window between the X1/X2 and the neo-Y was calculated as follows: *T* = *D*/2*μ*.

To infer the evolutionary history of the neo-Y chromosome, we calculated the divergence time for different Y chromosomal sections by calculating the genetic distance between the X1 and X2 chromosomes and the corresponding region on the neo-Y chromosome within a 100 kb sliding window. This analysis revealed five clusters of different ages (section A [SA]: 55 Ma, section B [SB]: 28 Ma, section C [SC]: 55 Ma, section D [SD]: 48 Ma, and section E [SE]: 48-0 Ma) (fig. 3 and supplementary table S9, Supplementary Material online). The average divergence time of the X1-syntenic arm is higher than that of the X2-syntenic arm, indicating that a pair of autosomes corresponding to proto-X1 initially differentiated into XY sex chromosomes. The X2-syntenic arm was added later, generating the neo-Y sex chromosome. Thus, the divergence of sex chromosomes initiated on the X1-syntenic arm in SA and SC, which correspond exactly to the ends of the inverted segment. These sections show a continuous highest level of divergence over their whole lengths, in agreement with the notion that the inversion initiated or accelerated the differentiation of the ancestral Y and X1.

Interestingly, in the center of the inverted region, there is a section of much lower divergence. The formation of an inversion loop during pairing of the proto-Y and proto-X may have maintained a certain level of recombination for some time until it ceased later. There is a region of divergence adjacent to SC outside of the inversion, which is considerably high, indicating the suppression of recombination. However, the divergence level is lower than those in SA and SC. Divergence levels continue to be high beyond the fusion point into the X2 homologous region, which may indicate that the entire SD region stopped recombining when the fusion of Y with X2 occurred. From SD toward the distal end (SE region), divergence gradually declines and reaches the level of autosomes only at the very tip of the chromosome. This is different from the steep border between SA and the pseudoautosomal region on the X1 homologous arm.

### Identification of Sex-Biased Genes

In total, we annotated 1,838 genes in the X1HDR and X2HDR of the neo-Y chromosome. To identify candidate genes involved in sex determination, gonad development, and sex-biased genes, we generated transcriptomes of gonads from six females and six males in the early gonad development stages (60 and 80 days post hatching). We detected 945 differentially expressed genes (DEGs) (supplementary fig. S12, Supplementary Material online), including 87 DEGs (fig. 5 and supplementary table S10, Supplementary Material online), which could only be detected in males, and no DEGs were specifically expressed in female gonads. Interestingly, of the 87 DEGs, we noted the *ruvbl1* (ruvB-like 1), *park7* (protein/nucleic acid deglycase DJ-1), and *vhl* (von Hippel-Lindau disease tumor suppressor) genes, which have a known function in spermatogenesis and cell differentiation (Makino et al. 1998; Wagenfeld et al. 1998; Welch et al. 1998; Ma et al. 2003). Only one (ID: Male_chrY_262) of the 87 DEGs is a Y^+^X^−^-gene, which is located in the inversion region with no clear functional annotation. Additionally, many other DEGs can be ascribed to functions in meiosis, cell division, and spermatogenesis or spermatocyte metabolism.

## Discussion

Fusions of a sex chromosome to an autosome are surprisingly frequent, leading to multiple sex chromosome systems. Multiple sex chromosome systems have been found in many fish species from various families and are often characterized by a large metacentric heteromorphic chromosome. These neo-Y chromosomes have been hypothesized to originate from a Robertsonian rearrangement between the original Y chromosome and the autosome (Kitano et al. 2009; Ross et al. 2009). Consequently, chromosome pairing in male meiosis occurs in a trivalent manner for the two types of previously observed chromosome associations: the end-to-end and the chiasmatic types (Uyeno and Miller 1971, 1972; Suzuki and Taki 1988; Saitoh 1989; Ueno and Kang 1992; Bertollo and Mestriner 1998). Starting from cytogenetic observations in *Allodontichthys hubbsi* (Uyeno and Miller 1971), which also has a neo-Y chromosome, and our genomic data, we infer a mechanism of meiotic pairing of sex chromosomes for the spotted knifejaw, which occurs by end-to-end association in a trivalent manner. Due to the high sequence divergence between the neo-Y chromosome over its whole X1 homologous region and the centromere near the X2 homologous region, pairing may be restricted to the pseudoautosomal region at the tip of X1 and the distal region of X2 (supplementary fig. S13, Supplementary Material online).

Recently, a whole-genome sequence analysis of the barred knifejaw (*O. fasciatus*) also tried to identify the origin of the neo-Y chromosome and compare the sequences and genes between the female X1X1X2X2 and male X1X2Y barred knifejaw. However, the researcher only assembled 23 chromosomes of the male *O. fasciatus* (22 autosomes and neo-Y) (Xiao et al. 2020). In our study, we managed to assemble the accurate haplotype of the nonrecombining region of the X1, X2, and Y chromosomes in addition to 22 autosomes. The X1 and X2 chromosomes in male spotted knifejaw have good collinearity and similarity to those in the female assembly. This means that the haploid chromosome number of male spotted knifejaw should be 25 (22 autosomes, X1, X2, and Y) (Xu et al. 2019). In our male assembly result, the haplotypes of X1X2 and Y were separated and confirmed using sex chromosome-specific SNPs, which ensured that the haplotype assembly was accurate. The two species (spotted knifejaw and barred knifejaw) are closely related and have similar karyotypes. Further comparative studies of spotted knifejaw and barred knifejaw sex chromosome structure could provide new insights into the evolution of the X1X2Y system.

**Fig. 4.**
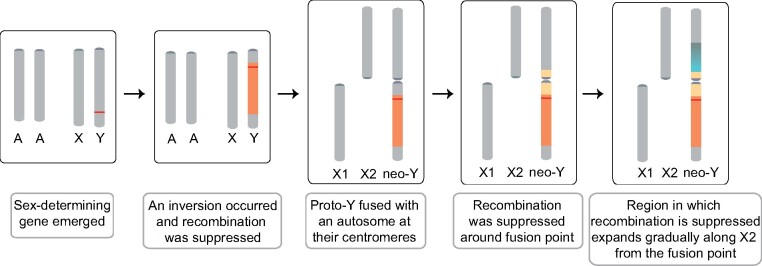
Model for the evolution of the Y chromosome in spotted knifejaw.

Our data allow us to infer the trajectory for the evolution of the neo-Y chromosome of spotted knifejaw (fig. 4). The X and ancestral Y chromosomes evolved from a pair of homologous autosomes. This process started over 60 Ma according to the high divergence times calculated for SC close to the centromere of the Y chromosome. Next, recombination was further suppressed by a large inversion that was estimated to have occurred ∼55 Ma and included SA, SB, and SC. Finally, a Robertsonian translocation fused the Y chromosome to the X2 chromosome, which we estimate at 48 Ma, leading to recombination suppression in the centromere near the X2HDR region. Based on this timing, the fusion of the Y chromosome to an autosome in knifejaw occurred approximately at least 46 Ma earlier than that of the Japanese stickleback and *D. miranda*, which were estimated to have occurred 1.5–2 and 1 Ma, respectively (Bachtrog and Charlesworth 2002; Yoshida et al. 2014). Although the recently added X2HDR in young neo-Y chromosomes is much different from the X1HDR in terms of sequence similarity to the corresponding region on the X chromosomes and in degree of degeneration, in the spotted knifejaw, half of the additional portion of X2 has features of high molecular differentiation that are more similar to the inversion segment on X1DHR rather than to the PAR.

**Fig. 5:**
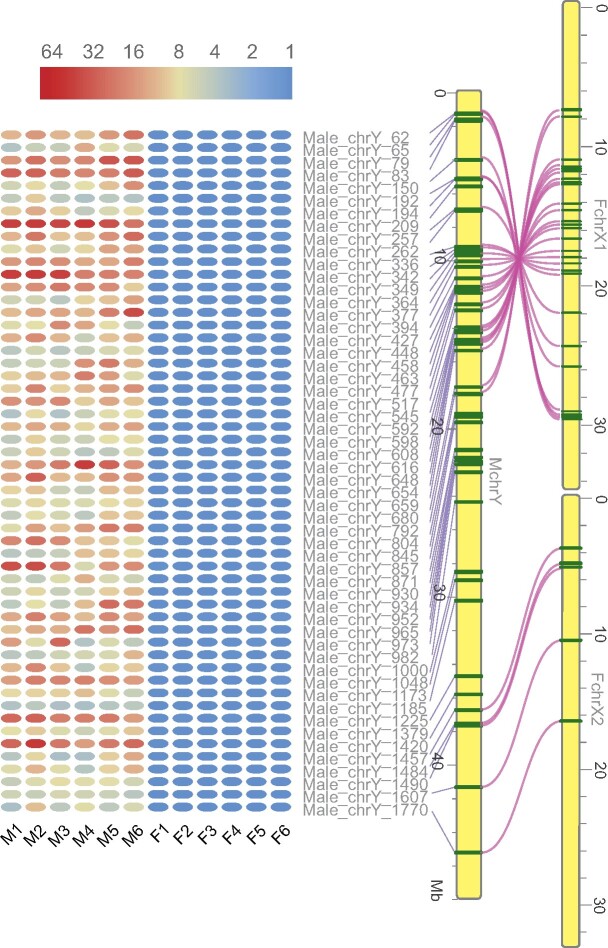
DEGs specifically expressed in male gonads based on the transcriptomic analysis. Fifty-four genes with gametologs on X chromosomes and the Y chromosome are shown. Forty-nine genes are in the inversion region (∼23 Mb) of the Y chromosome in X1HDR, whereas five are in the Y chromosome in X2HDR (∼17 Mb). The color bar shows the TPM values from 1 to 64. Red lines linking two chromosomes indicate the location of gametologs of the DEGs specifically expressed in the males.

The age of the spotted knifejaw neo-Y chromosome of ∼60 My makes it one the oldest sex chromosomes in fish characterized so far. At such old age, considerable genetic decay of the nonrecombining region is expected. Thus, it was surprising to find that its degeneration has progressed much less than in the similar fully characterized W chromosome of the tongue sole, which is only 30 My old and has a large inversion (Chen et al. 2014). The tongue sole W chromosome has already lost 70% of the 904 genes on the homologous region of the Z chromosome, whereas only 20% of genes were lost on the neo-Y chromosome of the knifejaw. In the same direction, sequence similarity is still high between the X1 and neo-Y chromosomes, the number of pseudogenes is increased only 2-fold on the neo-Y chromosome compared with 5-fold on tongue sole W chromosome, and the accumulation of transposons in non-PAR is only 1.5-fold, whereas it is 7-fold on the tongue sole sex chromosome. This provides molecular evidence for the reasoning that the degenerative processes of sex chromosome evolution run at diverse paces in different fish lineages.

We propose that the inversion was a key trigger during the emergence and further evolution of the neo-Y chromosome. It is reminiscent of the evolutionary scenario of an inversion that captures both a sex-determining mutation and a sex-antagonistic locus that was proposed for the Japanese three-spined stickleback (Peichel et al. 2004; van Doorn and Kirkpatrick 2007). The presence of sex beneficial genes on sex chromosomes has been assigned an important role in driving Y chromosome evolution (Rice 1987; Charlesworth et al. 2005; Kirkpatrick 2017). Transcriptomic analysis indicated a whole suite of highly diverged genes (86) and one Y^+^X^−^-gene on the neo-Y in the spotted knifejaw that are expressed in the testis, but not in the ovary. We hypothesize that this sex-biased expression suggests a “male-beneficial” role for these genes. Future comparative analyses with a related species where these genes are autosomal will allow us to infer if the sex-biased expression is a cause or consequence of sex-linkage.

In summary, the high-quality chromosome-size assembly of the spotted knifejaw male and female genomes, including the Y chromosome and both X chromosomes, allowed for the reconstruction of the evolutionary history of a vertebrate neo-Y chromosome and the analysis of its peculiar gene content for the first time. This study will provide the basis for further evolutionary and functional analyses to improve our understanding of the outstanding variety of sex chromosomes and sex-determining mechanisms, and how this plasticity evolved.

## Materials and Methods

### Sample Collection and Sequencing

Genomic DNA (for short insert library sequencing and 10X WGS) was extracted from the muscle of one female and one male spotted knifejaw separately, which were obtained from Laizhou Mingbo Fisheries Company Ltd. (Yantai, China). Genomic DNA was isolated and processed according to the DNA extraction protocol. We constructed 10X Genomics sequencing libraries using the 10X Genomics Chromium System with 15 µg DNA following the manufacturer’s protocol (Chromium Genome v1, PN-120229). The BGISEQ-500 platform was used to perform 2 × 150 paired-end sequencing. In total, we obtained 277.2 Gb (123.6 Gb for female and 153.6 Gb for male) of raw sequence data (supplementary table S11, Supplementary Material online). To reduce the effect of sequencing errors on the assembly, we used SOAPnuke to filter out low-quality reads with adaptors, high base error rate, and highly unknown base proportion and obtained 232.5 Gb of clean data.

DNA for nanopore sequencing was isolated from the muscle of the same male mentioned above using the QIAamp DNA Mini Kit (Qiagen) and sequenced on the PromethION platform using the R 9.4 nanopore. DNA isolation, library preparation, and sequencing on the PacBio Sequel II platform were conducted according to the manufacturer’s protocol. Finally, 73.2 and 130.3 Gb of raw data were obtained separately on the two sequencing platforms.

To prepare the Hi-C library, blood samples were fixed with formaldehyde, and the restriction enzyme (MboI) was added to digest the DNA, followed by repairing 5′ overhangs using a biotinylated residue. A paired-end library with an ∼300 bp insert size was constructed following the Hi-C library preparation protocol, which was available on protocols.io. We performed sequencing of the female and male Hi-C libraries using the BGISEQ-500 platform, where the read length for each end was 100 bp, and finally obtained a total of 299.5 Gb raw Hi-C data.

Seventy-three males and 124 females of mature spotted knifejaw were sampled from Laizhou Mingbo Fisheries Ltd Company, Yantai, China, for whole-genome resequencing. Genomic DNA was isolated and processed as described above.

We collected RNA from 12 gonad samples at 2 developmental stages (60 and 80 days post hatching), with three males and three females in each stage. All RNA of these samples was extracted by the TRIzol reagent (Invitrogen, Carlsbad CA, USA) according to the manual instructions and treated with RNase-free DNase l (TaKaRa, Dalian, China) to degrade residual DNA. Then, the cDNA was transcribed with 1 μg of total RNA using the Reverse Transcriptase M-MLV kit (TaKaRa) following the protocols. Then, all 12 transcriptomes were sequenced on the BGISEQ500 platform and filtered by SOAPnuke with the parameters of “-M 1 -A 0.4 -n 0.05 -l 10 -q 0.4 -Q 2 -G -5 0,” generating 187 Gb of clean data.

The genetic sex of all the fish used in this study was identified using male-specific DNA markers from spotted knifejaw as previously described (Li et al. 2020), and the phenotypic sex of the fish was identified using histological sectioning and HE staining as previously described (Li et al. 2020).

### Genome Assembly

For the female genome assembly, we employed SUPERNOVA v2.0 using clean 10X Genomics data for initial assembly with default parameters as follows: build a 48-mer DBG based on shared *k*-mers, map barcodes to the de Bruijn graph, use barcode information to scaffold, partition the graph and make local assembly, phase based on barcode information, close gap, and reuse barcode and copy number information to further scaffold. GAPCLOSER v1.12 (Luo et al. 2012) was used to reduce gap regions. Quality control of Hi-C raw data was conducted using HiC-Pro v2.8.0 (Servant et al. 2015). First, Bowtie2 v2.2.5 (Langmead et al. 2009) was used to align the reads to the gap-closed sequences; high-quality reads were taken to build raw inter/intra-chromosomal contact maps. Then, we used Juicer v1.5 (Durand et al. 2016) to prepare Hi-C data with valid pairs reads and 3DDNA v170123 (Dudchenko et al. 2017) to elevate the assembly to chromosome levels. More detailed pipeline about scaffolding using Hi-C data is as described on protocol.io (dx.doi.org/10.17504/protocols.io.qradv2e, last accessed March 3, 2021). For the male genome, we used SUPERNOVA with 10X Genomics for initial assembly, then GAPCLOSER with short reads to fill gaps and TGS-GapCloser v1.0.0 (Xu et al. 2020) with Nanopore reads to rescaffold using default parameters, and we finally used 3DDNA with Hi-C data to elevate the assembly to chromosome levels. The genome quality was evaluated by BUSCO v3.0 based on 4584 genes of Actinopterygians. However, for the male, we could first construct only 24 chromosomes, which is inconsistent with the number of karyotypes (25).

### Genome Annotation

For repetitive element predictions, RepeatMasker v3.3.0 (setting -nolow -norna -no_is) (Tarailo-Graovac and Chen 2009) and RepeatProteinMask v3.3.0 (setting -engine ncbi -noLowSimple -*P* value 1e−04) were used to perform predictions based on homologous sequences in RepBase v17.01 (Bao et al. 2015). The LTR_FINDER v1.05 (Xu and Wang 2007) and TRF v4.04 (setting Match = 2, Mismatch = 7, Delta = 7, PM = 80, PI = 10, Minscore = 50, MaxPeriod = 500) (Benson 1999) tools were used for de novo prediction based on the features of the repeat sequences.

The genome structure analysis was conducted using homology-based prediction, transcriptome-based prediction and de novo prediction. For homology annotation, we selected eight teleost species, including *O. fasciatus*, *Astyanax mexicanus*, *Cynoglossus semilaevis*, *Danio rerio*, *Gadus morhua*, *Gasterosteus aculeatus*, *Larimichthys crocea*, *Lepisosteus oculatus*, *Takifugu rubripes* and *Tetraodon nigroviridis*, and downloaded their protein sequences from the National Center for Biotechnology Information (NCBI) database. These protein sequences were aligned to the genome assembly by Blast with an E-value cutoff of 1e−5. The best hits were linked by SOLAR (Li et al. 2010), and the exact gene structures were defined by GENEWISE v2.4.0 (Birney et al. 2004). For the transcriptome-based annotation, we first assembled the transcriptome by TRINITY v2.1.1 (Grabherr et al. 2011) with RNA-seq data, then used PASA (Haas et al. 2003) to make alignments to the genome, and finally used Transdecoder (https://github.com/TransDecoder/TransDecoder/wiki, last accessed March 3, 2021) to identify and obtain ORFs. For the de novo prediction, the gene structures were analyzed on the repeat-masked genome assembly using AUGUSTUS v2.5.5 (Stanke and Waack 2003), GLIMMERHMM v3.0.4 (Majoros et al. 2004) and GENSCAN (Burge and Karlin 1997) with default settings. Of these, AUGUSTUS was trained by 1500 gene models of transcriptome-based annotation results. Finally, EVidenceModeler (Haas et al. 2008) was used to integrate the three evidence sets.

Pseudogenes were identified using the method described in the tongue sole study (Chen et al. 2014). In short, the genes in the homology-based gene prediction section were regarded as pseudogenes if they contained more than two frame errors (frameshift or internal stop codons) for multiple-exon genes. Finally, 19393 genes and 714 pseudogenes were predicted in the homology-based gene set.

For the functional annotation of the gene sets, the NR, KEGG (Kanehisa and Goto 2000), SwissProt, and Trembl (https://www.uniprot.org/statistics/TrEMBL, last accessed March 3, 2021) databases were searched to identify homologous proteins using Blastp with an E-value cutoff of 1E-5 (Kanehisa and Goto 2000; Bairoch et al. 2005). InterProScan v4.7 (Jones et al. 2014) was employed to obtain protein domain annotation and Gene Ontology annotation (Mitchell et al. 2019).

### Resequencing and SNP Calling

Clean reads of 73 males and 124 females were obtained by filtering out low-quality reads using SOAPnuke as mentioned above and aligned to the female reference genome using BWA with default parameters. Then, Picard v.1.105 was used to sort the alignment files (bam) and mark the duplicate reads. We used GATK v.4.0 to analyze all bam files and generate SNP files. Finally, we used VCFTOOLS v1.15 (Danecek et al. 2011) to filter the vcf files with the parameter of “-max-alleles 2 -min-alleles 2 -maf 0.05 -max-missing 0.8.”

### Genome-Wide Association Study

The cMLM of GAPIT v2 (Tang et al. 2016) was used to carry out the GWAS on the sequencing data mentioned above. The cMLM model can deal with bias caused by population structure and familial relatedness (kinship) and can be described as *Y* = *Xβ* + *Zu* + *e*, where *Y* represents the phenotype, vector *β* is the fixed effects of population structure (*Q*), vector *u* explains the random effects of kinship (*K*) among individuals, vector *e* is unobserved factors, *X* and *Z* are Henderson’s matrices related to *β* and *u*.

### *F*_ST_, Nucleotide Diversity, and Heterozygosity Analyses

VCFTOOLS was used for *F*_ST_, nucleotide diversity, and heterozygosity analyses with the SNP data of 73 males and 124 females. We carried out genome-wide *F*_ST_ analysis within a 100 kb sliding window. The nucleotide diversity of females and males was calculated within a 100 kb sliding window. We also calculated the observed heterozygosity of every individual in different regions. Student’s *t*-test was performed to detect whether there was a significant difference between males and females in chrX1 and chrX2 as well as other regions.

### Male Genome and Neo-Y Assembly

We first identified the sex chromosome-specific SNPs (scsSNPs) in the divergent regions of the sex chromosomes (X1 and X2 of the female assembly result) using resequencing data from the 197 individuals mentioned above. SNPs are considered to be scsSNPs only if they showed female homogamety and male heterogamety. Then, the contigs of the male assembly result (CANU v1.8: genomeSize = 850 m min ReadLength = 1,000 corOutCoverage = 120 stopOnReadQuality = false) (Koren et al. 2017) using PacBio data were characterized by the scsSNPs after being aligned to the female assembly using minimap2 (Li 2018). SNPs were called using utilities (paftools.js) in minimap2. The contigs were confirmed to be Y- or X-related only if they had exclusive Y- or X-specific SNPs. Furthermore, the other three assembly versions (CANU v1.8: genomeSize = 800 m minReadLength = 1,500 corOutCoverage = 40; CANU v1.8: genomeSize = 800 m minReadLength = 500 corOutCoverage = 80; Falcon v1.3.0: genome_size = 850,000,000) (Chin et al. 2013; Koren et al. 2017) of the PacBio reads were used to merge these divided contigs. However, it was impractical to directly assemble the low divergence region of X2 (SE in fig. 4). We used HapCut2 v1.2 (Edge et al. 2017) to haplotype this region in a sliding window of 6 M and a step size of 1 M to avoid switch errors. Then, the PacBio raw reads were separated into two haplotypes and assembled, respectively, using CANU (CANU v1.8: genomeSize = 6 m minReadLength = 1,500 corOutCoverage = 40) in each bin. Then, the assembled contigs were separated and confirmed using scsSNPs as mentioned above. Finally, they were manually scaffolded using the overlapping sequence of neighbor bins with Mummer v4.0beta (Marcais et al. 2018).

### Identification of Y^+^X^−^-genes in the Inverted Region

The genes on the Y, which have no homologous sequences on the X1-syntenic arm are named as Y^+^X^−^-genes. To identify the Y^+^X^−^-genes in the inverted region, MCscan (Wang et al. 2012) was first employed to identify synteny blocks and homologous gene pairs between Y and X based on the gene set. Then software Flo (LiftOver) (Pracana et al. 2017) was used to further determine whether one Y gene had a homolog on the X. Only if a Y gene was marked with “Deleted” by software Flo, it was regarded as Y^+^X^−^-gene candidate. Last, these genes were further taken as gene models to annotate the X chromosome through the homology-based method as described above and those genes with no annotation result were kept and regarded as Y^+^X^−^-genes.

### Divergence Time between Chromosome X1/X2 and Y

We estimated the nucleotide mutation rate through comparative analysis between spotted knifejaw and ten other species, including *O. fasciatus*, *A. mexicanus*, *C. semilaevis*, *D. rerio*, *G. morhua*, *G. aculeatus*, *L. crocea*, *L. oculatus*, *T. rubripes*, and *T. nigroviridis*. First, we used BlastP with an *E*‐value threshold of 1e−07 and TreeFam (Li et al. 2006) to compare protein sequences between each other and to generate orthology and paralogy relationships among all the species. Then, the single-copy orthologs were used to construct phylogenetic trees by PhyML with parameters of “-m F84, -a invgamma, -b -2.” The divergence time between the spotted knifejaw and others was estimated by MCMCTREE (Yang 1997) according to the fossil time (42–57 Ma between *T. rubripes* and *T. nigroviridis*, 99–127 Ma between *O. fasciatus* and *L. crocea*) from TIMETREE (http://timetree.org/, last accessed March 3, 2021) (supplementary fig. S14*b*, Supplementary Material online). The genetic distance between the spotted knifejaw and the other species was calculated by DISTMAT (EMBOSS : 6.5.7.0) with the “Kimura” nucleotide substitution model and default parameters. Finally, the estimated mutation rate of the spotted knifejaw was ∼8.18 × 10^−10^.

We used LASTZ v0.9 to compare the sequence collinearity between the female X1/X2 chromosomes and the male neo-Y chromosome. Then, we calculated the genetic distance of blocks larger than 1,000 bp within a 100 kb sliding window. The divergence time of each window between the X1/X2 chromosomes and neo-Y chromosome was calculated according to the following formula: *T* = *D*/2*μ*, where *T* represents divergence time, *D* means the genetic distance, and *μ* is the nucleotide mutation rate assumed to be equal to the rate of 8.18 × 10^−10^.

We also estimated X-Y divergence using Ks-based (*r* = Ks/2*t*) methods. The divergence time of spotted knifejaw and striped knifejaw was predicted to be 11.5 Ma (5.6–20.9). The Ks values of homolog pairs between spotted knifejaw and striped knifejaw were calculated using KaKs_calculator (ParaAT2.0) with the gene pairs of the single-copy gene family as input. The Ks value 0.03 corresponding to the peak of the Ks distribution was used for further analysis. Therefore, the *r* value here was ∼1.3 × 10^−9^ (0.03/(2 × 11.5 × 10^−6^)). The Ks values of homolog pairs between Xs and Y were also calculated using KaKs_calculator. The mean Ks value of gene pairs in SA, SB, and SC was ∼0.157. The divergence time is shown in supplementary figure S15, Supplementary Material online. The trend of the divergence time is nearly the same as that in figure 3. However, the mean divergence time calculated using the Ks method is slightly shorter than that shown in figure 3. The result in figure 3 may reflect the evolution of the whole non-PAR including coding and noncoding sequences, although it might be slightly overestimated. To understand the trajectory for the evolution of the neo-Y chromosome of spotted knifejaw macroscopically, we mainly referred to the divergence time calculated using the DNA sequences in the result and discuss part.

### Differentially Expressed Genes

All steps of RNA-Seq analysis were conducted through the RNACocktail pipeline (Sahraeian et al. 2017). HISAT2 v2.1.0 was used to align all sequences of 12 samples to the female reference genome and the male reference genome separately. Then, the transcripts per million (TPM) of each sample was calculated by SALMON v0.14.1. Genes are regarded as DEGs specifically expressed in male gonads only if their TPM values are equal to zero in at least two female ovaries (<0.1 in other ovaries) and >1 in male testes.

### Polymerase Chain Reaction Validation of DEGs

To verify the male-specific sequences obtained above based on RNA-seq data, two primer pairs were designed using Primer5 software.

Total RNA was extracted from the gonads of five females and five males sampled on the 50th day post hatching by Trizol reagent and treated with RNase-free DNase (TaKaRa, Dalian, China) according to the manufacturer’s instructions. First-strand cDNA was synthesized using AMV reverse transcriptase (Takara, Dalian, China) with oligo d(T) primer according to the manufacturer’s instructions. The obtained cDNAs were used for polymerase chain reaction (PCR).

The cycling conditions were 3 min at 95 °C; 30 cycles of 95 °C for 30 s, 55 °C for 30 s, and 72 °C for 7 min; and, last, incubation at 72 °C for 7 min. All PCR products were analyzed on 1% agarose gels by agarose gel electrophoresis.

Two genes (*dtnbp1*, dysbindin, ID: Male_chrY_934 and *ptrh2*, peptidyl-tRNA hydrolase 2, ID: Male_chrY_1067) of the DEGs were confirmed using PCR and agarose electrophoresis with one band in the five testis samples and no band in the five ovary samples on the 1% agarose gel (primers: supplementary table S12, Supplementary Material online; electropherogram: supplementary fig. S16*a*, *b*, Supplementary Material online).

### Code Availability

No specific code was developed in this study. The data analyses were conducted following the manuals or protocols provided by the developers of the corresponding bioinformatics tools that are described in the Materials and Methods section. Bioinformatic tools were run with default parameters except for those specified.

## Supplementary Material

Supplementary data are available at *Molecular Biology and Evolution* online.

## Supplementary Material

msab056_Supplementary_Data
