# Supplementary material for: Reconstruction of the Origin of a Neo-Y Sex Chromosome and Its Evolution in the Spotted Knifejaw, Oplegnathus punctatus
Source: Mol Biol Evol. 2021 Mar 9;38(6):2615–26. doi: 10.1093/molbev/msab056 (PMC8136494; doi:10.1093/molbev/msab056)
Supplement: msab056_Supplementary_Data [file msab056_supplementary_data.zip › Supplementary Fig. S13.pdf]

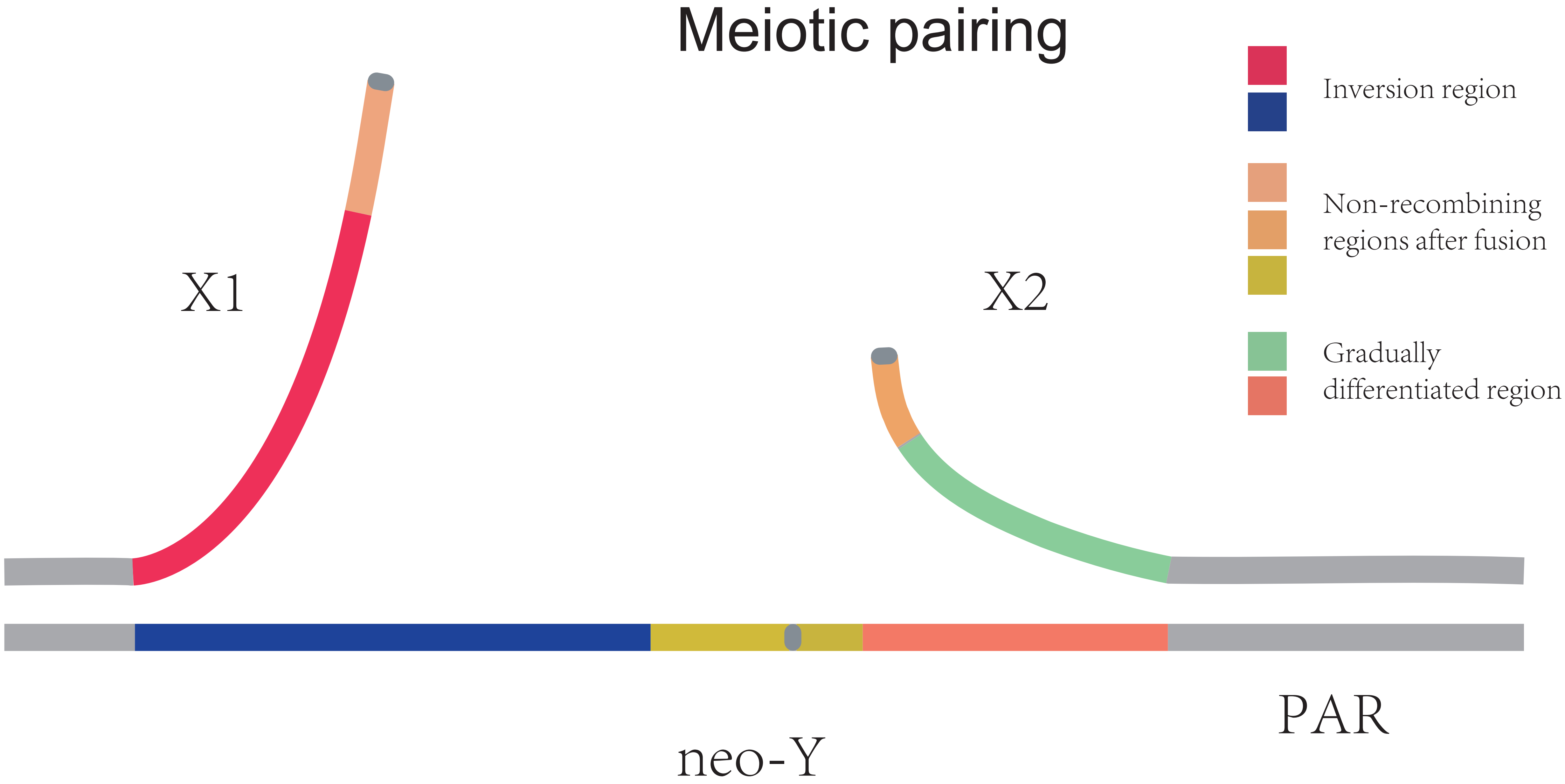
Meiotic pairing
Inversion region
Non-recombining regions after fusion
Gradually differentiated region
X1
X2
PAR
neo-Y
