# Supplementary material for: Reconstruction of the Origin of a Neo-Y Sex Chromosome and Its Evolution in the Spotted Knifejaw, Oplegnathus punctatus
Source: Mol Biol Evol. 2021 Mar 9;38(6):2615–26. doi: 10.1093/molbev/msab056 (PMC8136494; doi:10.1093/molbev/msab056)
Supplement: msab056_Supplementary_Data [file msab056_supplementary_data.zip › Knifejaw_supplementary_materials_MBE--for_First_look.pdf]

Supplementary Materials for

Reconstruction of the origin of a neo-Y sex chromosome and its evolution in the spotted knifejaw, *Oplegnathus punctatus*

This PDF file includes:

Supplementary Figures 1 to 16
Supplementary Tables 1 to 13

Supplementary Figures

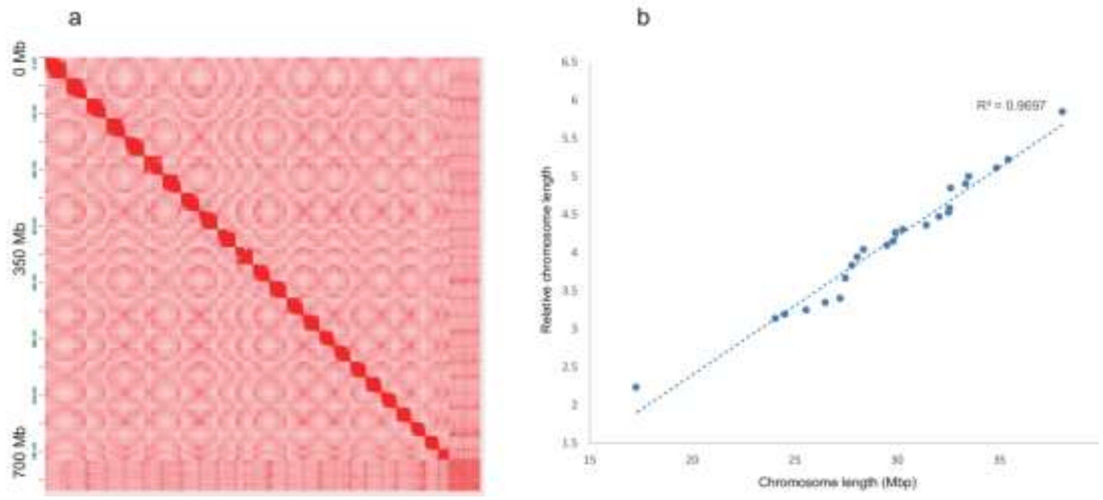

Fig. S1 Chromosome-level assembly of the female spotted knifejaw genome using Hi-C data. (a) Heatmap of contact metrics generated from mapping of Hi-C reads to genome sequences. (b) Correlation between Hi-C assembled chromosome length and physical length (Li, et al. 2016).

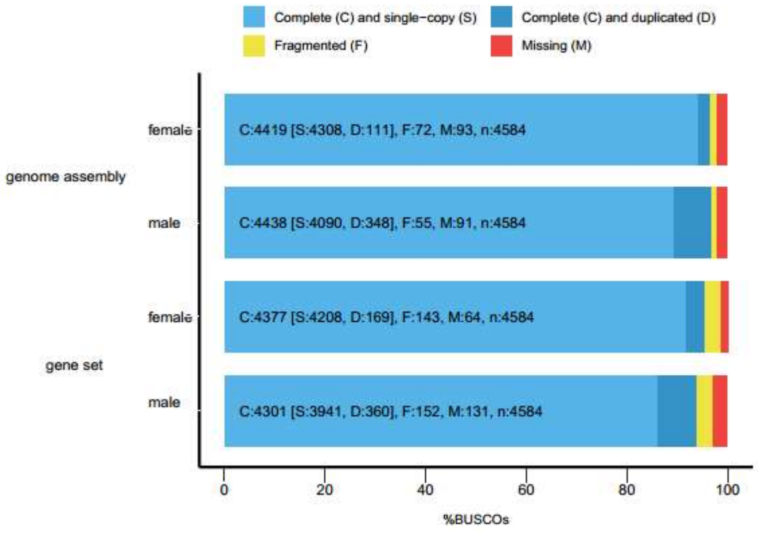

Fig. S2 Genome assembly evaluation. The BUSCO dataset of the Actinopterygii odb9 including 4,104 BUSCOs was used to assess the genome assembly and gene set of female and male spotted knifejaw.

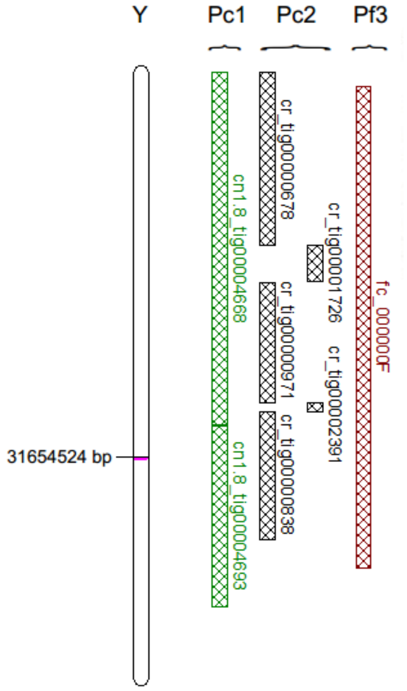

Fig. S3 Assembly of the high divergence region of the neo-Y chromosome. Y contigs from three genome assembly versions (Pc1, Pc2 and Pf3) were merged into one contig. The centromeric region is indicated by a pink line.

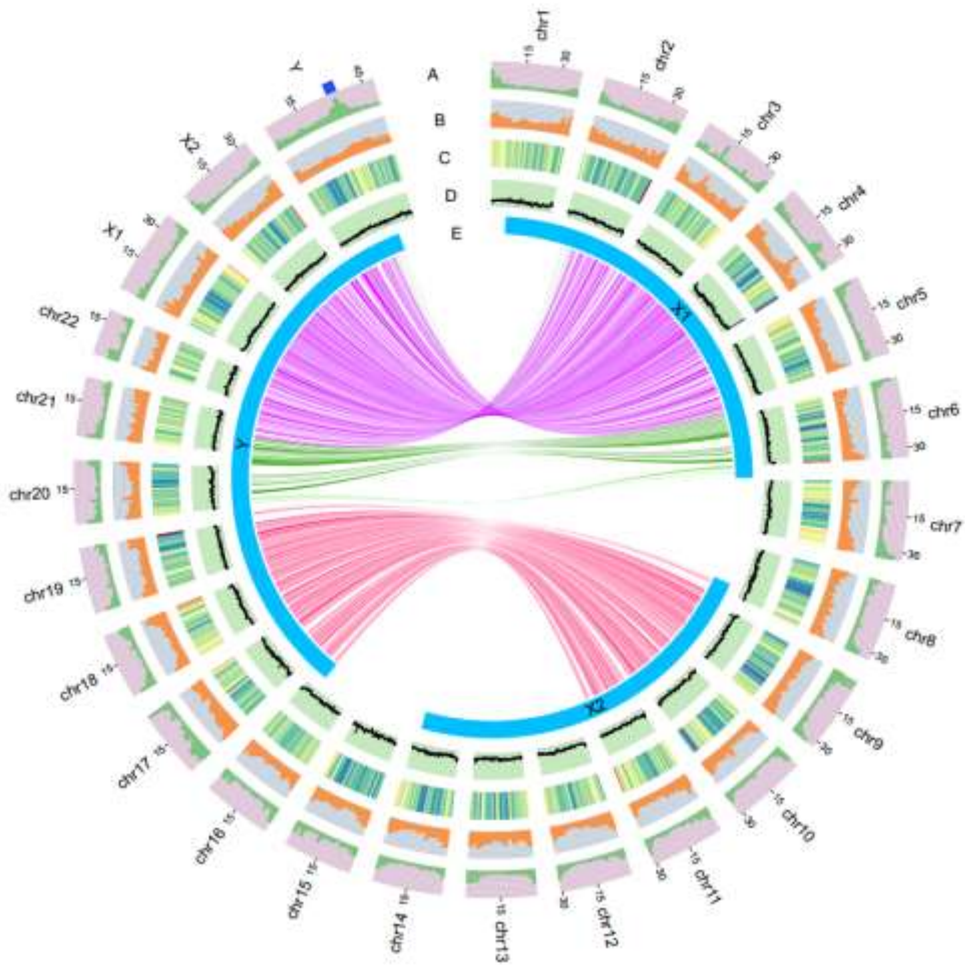

Fig. S4 Genomic features of the male spotted knifejaw. From outer to inner circles: A, distribution of DNA transposons across the genome; B, distribution of retrotransposons across the genome; C, gene density; D, GC content across the genome; E, represents three sex chromosomes. In the inner, each line joins gametologs between the X chromosomes and the Y chromosome. The predicted fusion region on the Y chromosome is indicated with one dark blue bar at the outermost circle.

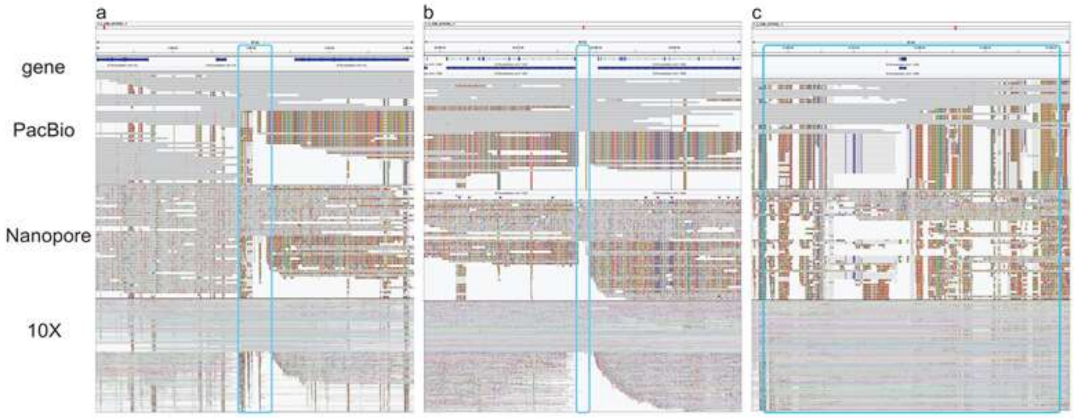

Fig. S5 Confirmation of the inversion and fusion points on the neo-Y chromosome. Visualization of the alignment of the PacBio, Nanopore and 10X genomics reads to the neo-Y chromosome with IGV. (a) and (b) show the regions around the start and end points of the inversion, while (c) shows the region of the fusion point.

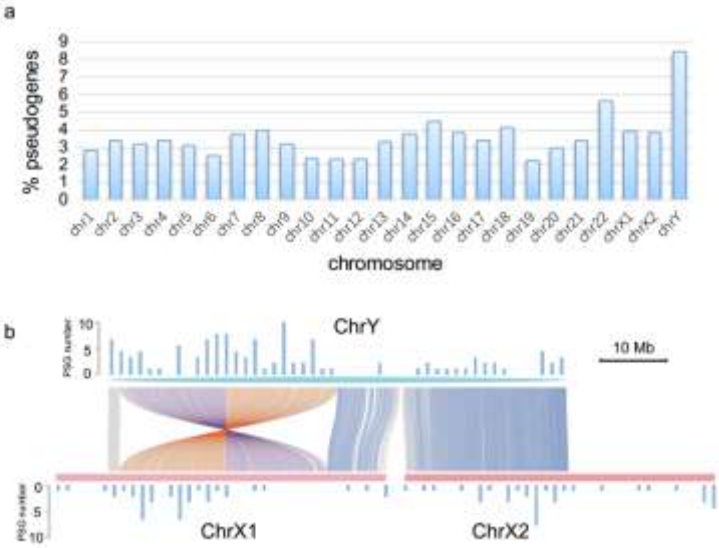

Fig. S6 Distribution of pseudogenes on different chromosomes. (a) Distribution of pseudogenes on all chromosomes. (b) Distribution of pseudogenes on sex chromosomes. The bar charts near the chromosomes show the number of pseudogenes (PSG number) in a window of 1Mb with a maximum of 10 genes. The synteny relationships are described in the legend of Fig. 1. The pseudogenes were identified using the method described in (Chen, et al. 2014). In short, the genes in the homology-

based gene prediction section were regarded as pseudogenes if they contained frame shifts. In total, 19393 genes and 714 pseudogenes were predicted in the homology-based gene set.

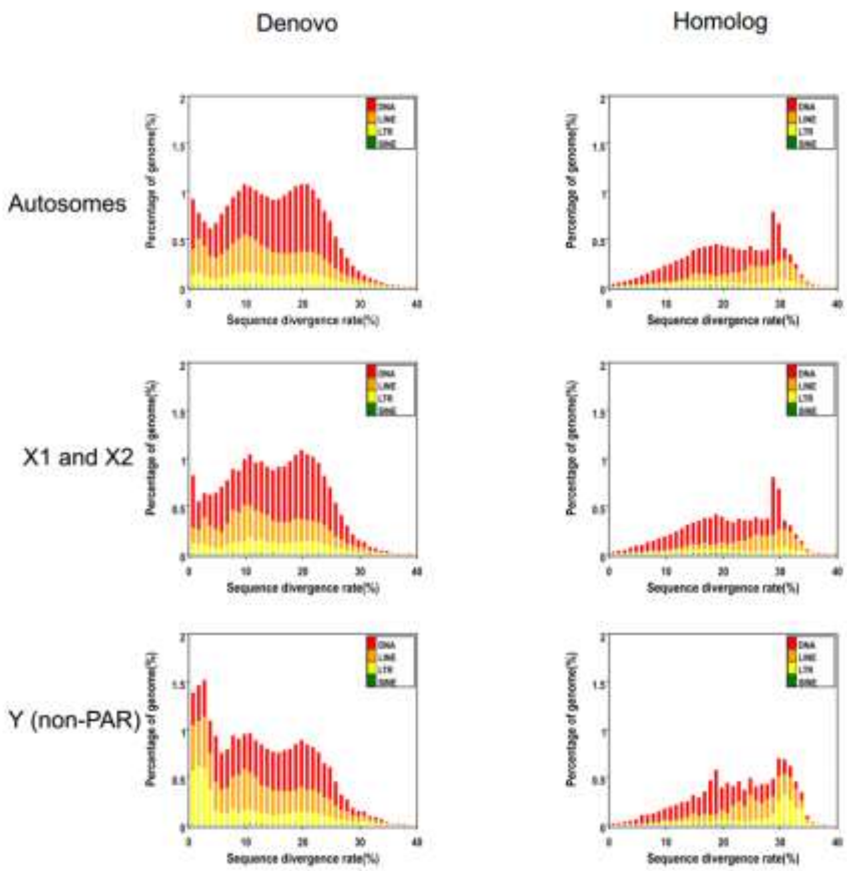

Fig. S7 The distribution of sequence divergence rates of transposable elements (TEs) as percentages of the size of the autosomes and sex chromosomes. "Denovo" means that the repeat sequences are de novo predicted using RepeatModeler, while "Homolog" means that the repeat sequences are predicted with homologs in Repbase using RepeatMasker.

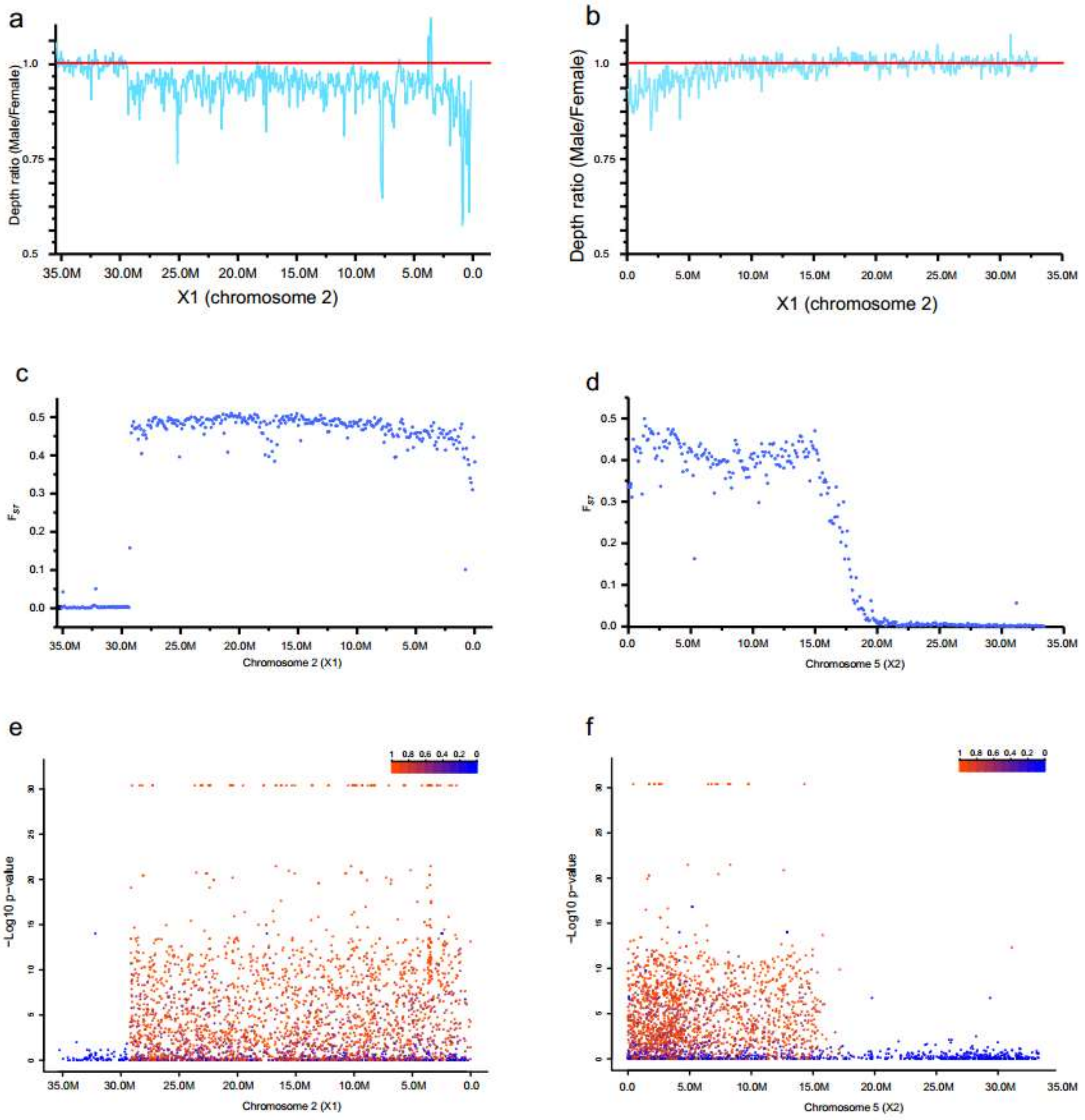

Fig. S8 Depth ratio analysis, F_{ST} analysis and Manhattan plot of the X1 and X2 chromosomes of females. (a) and (b) Average sequencing depth ratio between females and males. (c) and (d) Fixation index (F_{ST}). (e) and (f) Manhattan plots of X1 and X2. The X-axis represents the genomic position of the SNPs in the genome, and the Y-axis is the negative log base 10 of the *P*-values. Y-coordinate values larger than 30 are all scaled to 30. GWAS was performed using the compressed Mixed Linear Model implemented in GAPIT v2 (Tang, et al. 2016).

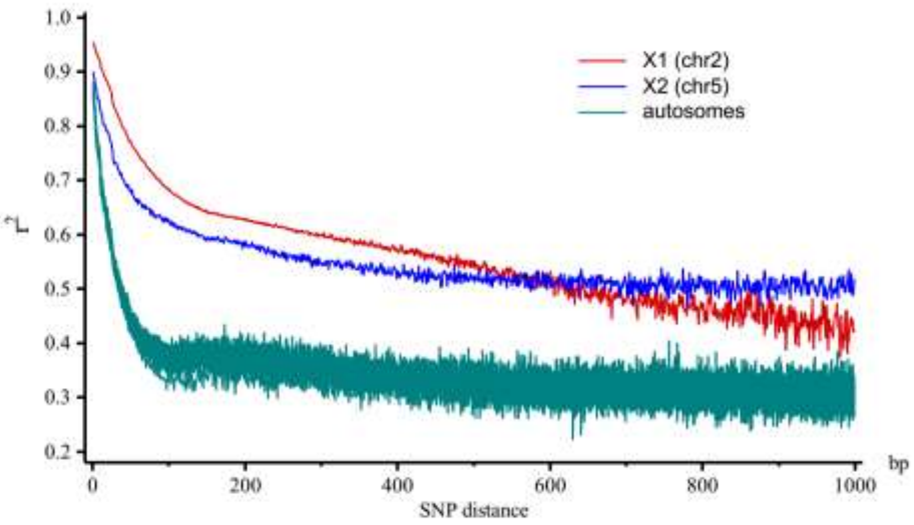

Fig. S9 SNPs on the X1 and X2 chromosomes constitute a linkage disequilibrium (LD) block. The average genotypic association coefficient r^2 (y-axis) is presented as a function of inter-SNP distance.

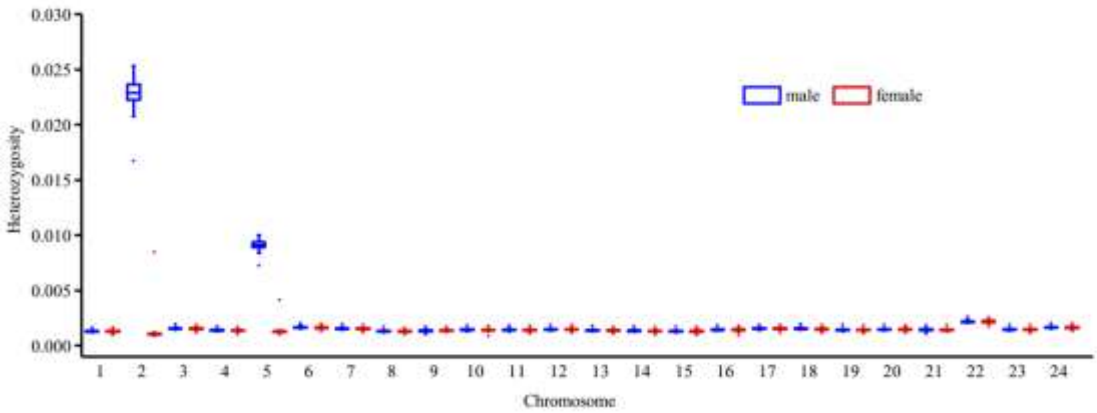

Fig. S10 Observed heterozygosity on X1 (chr2), X2 (chr5) and other chromosomal regions in spotted knifejaw males and females. Heterozygosity is depicted on the y-axis, and chromosomes are lined up on the x-axis.

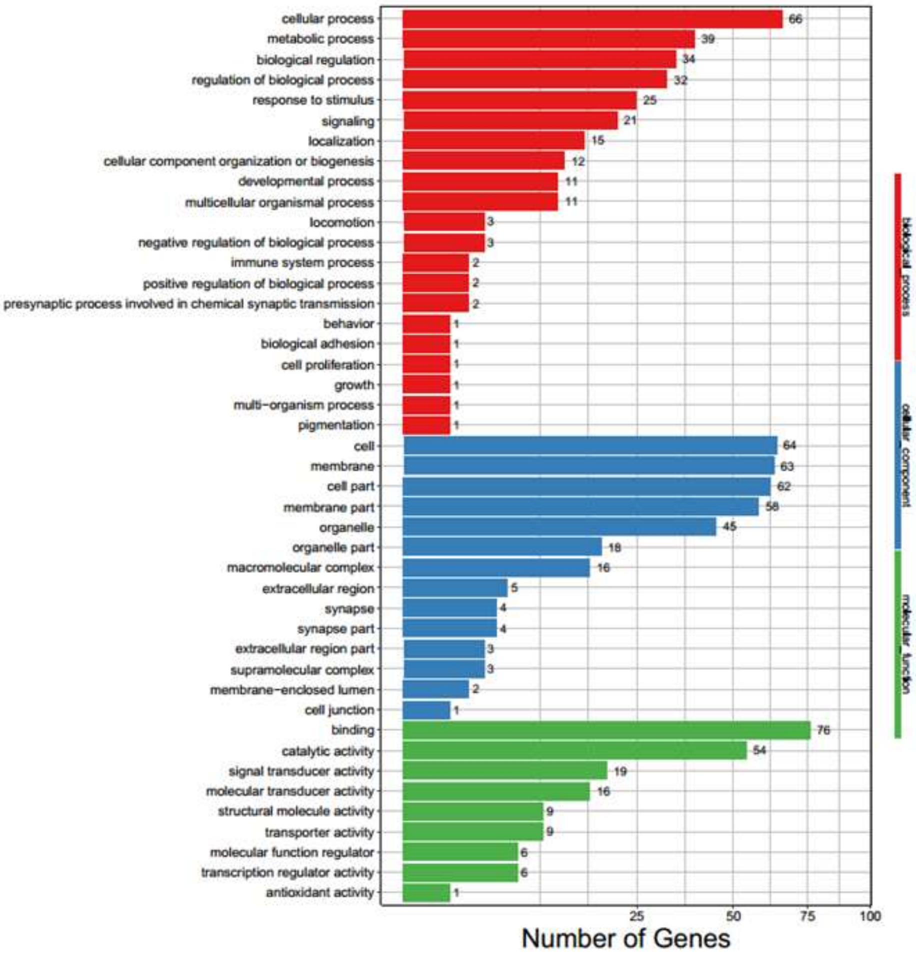

Fig. S11 GO classification of genes in the inversion region of neo-Y.

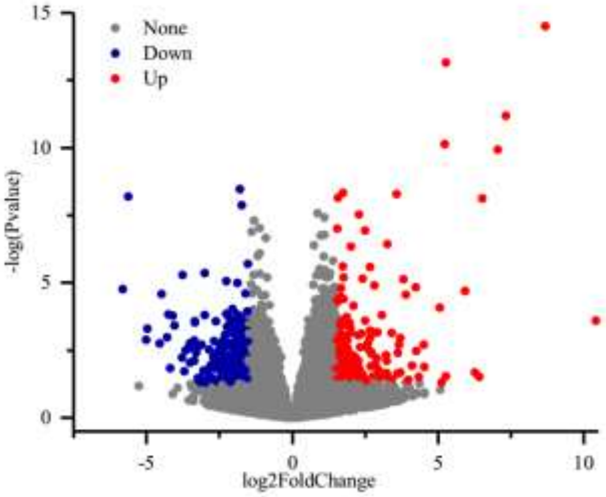

Fig. S12 Differentially expressed gene analysis in gonads between females and males. Volcano plot shows upregulated (FC > 2) and downregulated (FC < 0.5) genes

in red and green dots, respectively.

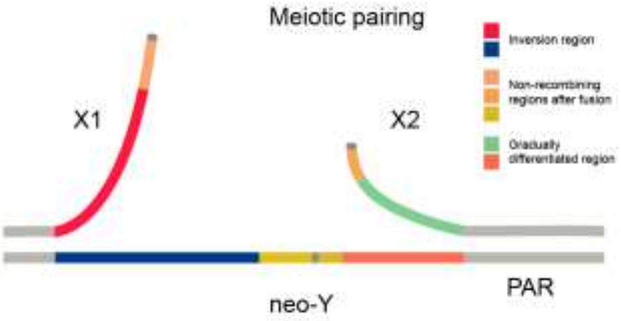

Fig. S13 Meiotic pairing.

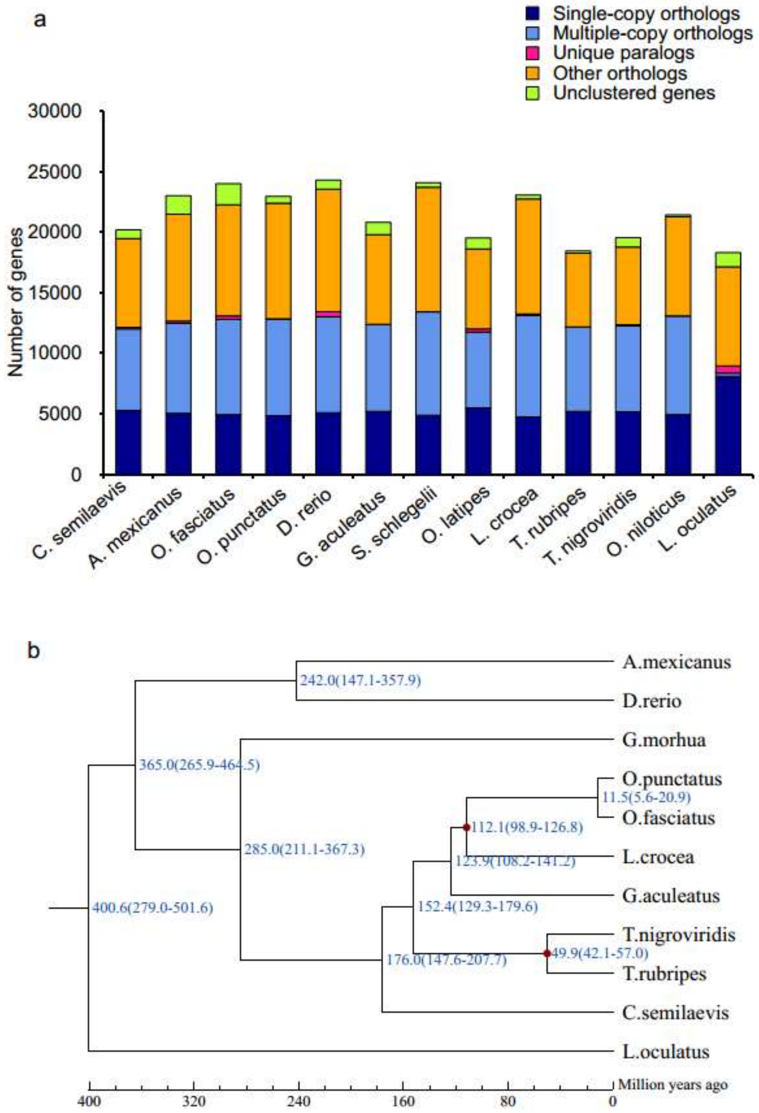

Fig. S14 Genome evolution analysis. (a) Identification of single-copy gene families. (b) Phylogenetic tree of 11 vertebrate genomes, which was constructed using single-copy orthologous genes. Divergence times (red circles) of *Takifugu rubripes* and *Tetraodon nigroviridis*, *Oplegnathus fasciatus* and *Larimichthys crocea* from the TimeTree database were used for calibration. The blue numbers show the estimated diverge times in millions of years ago.

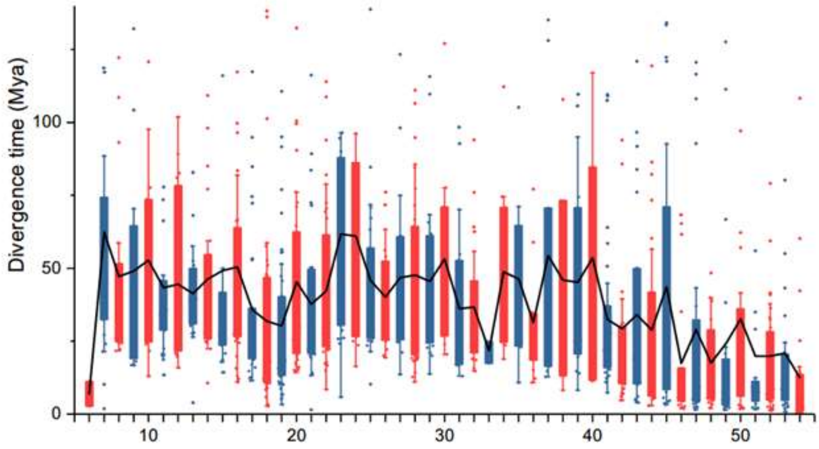

Fig. S15 Divergence times along the neo-Y chromosome in a sliding window of 100 kb. Divergence times were calculated using the Ks method.

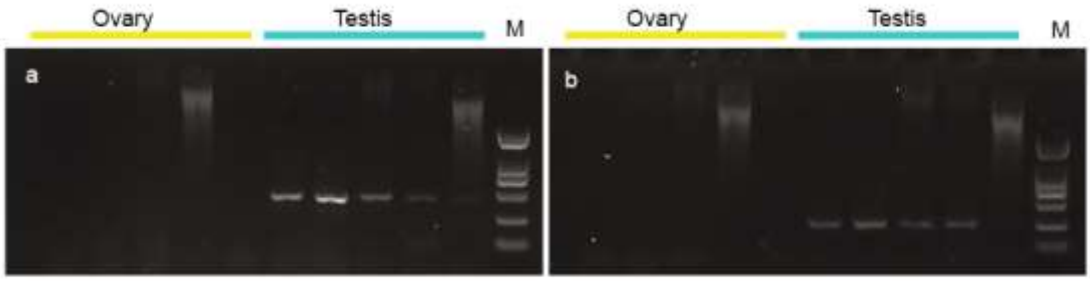

Fig. S16 PCR validation of DEGs. (a) and (b) Electrophoregram of the PCR products using the primers 934L/934R (left; gene id: Male_chrY_934; dtnbp1, dysbindin;) and 1067L/1067R (right; gene id: Male_chrY_1067; ptrh2, Peptidyl-tRNA hydrolase 2). The DNA marker is indicated by the letter M.

Supplementary Tables

Table S1 Summary statistics of genome assemblies.

Statistical level	Female		Male	
	Scaffold	Contig	Scaffold	Contig
Total length (bp)	758,615,438	732,813,513	831,432,090	827,276,721
Average length (bp)	31187.94	19909.08	44234.52	39048.27
N50 length (bp)	29,922,089	122,596	29,978,252	1,554,994
N90 length (bp)	24,090,098	15,842	17,662,374	127,926
Maximum length (bp)	38,061,106	1,057,150	49,798,034	49,798,034
GC content is (%)	40.85	40.85	40.85	40.85

Table S2 Assessment of female and male genome assemblies and gene sets with the BUSCO tool (using the dataset of the Actinopterygii odb9).

	Female		Male	
	Genome assembly	Gene set	Genome assembly	Gene set
Complete BUSCOs (C)	4419	4377	4438	4301
Complete and single-copy BUSCOs (S)	4308	4208	4090	3941
Complete and duplicated BUSCOs (D)	111	169	348	360
Fragmented BUSCOs (F)	72	143	55	152
Missing BUSCOs (M)	93	64	91	131
Total BUSCO groups searched	4584	4584	4584	4584
Total	C:96.4% [S:94.0%, D:2.4%]	C:95.5% [S:91.8%, D:3.7%]	C:96.8% [S:89.2%, D:7.6%]	C:93.9% [S:86.0%, D:7.9%]

Table S3 Alignment length of 22 autosomes between females and males.

Female Chromosomes				Alignment Length	Coverage
Num.	Name in assembly	Name(in Fig 1. a)	Length		
1	chr1	Fchr1	36966080	35413947	0.958
2	chr3	Fchr2	33561574	31610926	0.942
3	chr4	Fchr3	32381609	31758336	0.981
4	chr6	Fchr4	31285169	27098463	0.866
5	chr7	Fchr5	31422208	30643930	0.975
6	chr8	Fchr6	31554306	30552179	0.968
7	chr9	Fchr7	30857940	30154840	0.977
8	chr10	Fchr8	30520170	29469784	0.966
9	chr11	Fchr9	29332488	28073241	0.957
10	chr12	Fchr10	28966546	27746643	0.958
11	chr13	Fchr11	28824950	28218294	0.979
12	chr14	Fchr12	28472746	27706766	0.973
13	chr15	Fchr13	27452658	26981392	0.983
14	chr16	Fchr14	26875624	25754965	0.958
15	chr17	Fchr15	26515172	25143034	0.948
16	chr18	Fchr16	26559347	25042511	0.943
17	chr19	Fchr17	26262214	25277093	0.962
18	chr20	Fchr18	25264930	24196302	0.958
19	chr21	Fchr19	24706271	24005630	0.972
20	chr22	Fchr20	23248742	21645365	0.931
21	chr23	Fchr21	23171085	22150309	0.956
22	chr24	Fchr22	16743870	15972546	0.954
Total			687278860	660270554	0.961

Table S4 Distribution of pseudogenes predicted in the homology-based gene annotation section.

Chromosome	Functional genes	Pseudogenes	Pseudogenes %
X1	1053	41	3.89
X2	990	38	3.84
Y (non-PAR)	1303	110	8.44
Autosomes	16047	525	3.27

Table S5 Prediction of repeat elements in the male spotted knifejaw.

Type	Repeat Size	% of genome
Trf	19161656	2.292845
Repeatmasker	66856408	7.999902
Proteinmask	22881095	2.737905
De novo	205856222	24.63234
Total	240270829	28.75032

Table S6 Statistics of repeat elements in the male spotted knifejaw.

Type	Repbase TEs		TE proteins		De novo		Combined TEs	
	Length (Bp)	% in genome	Length (Bp)	% in genome	Length (Bp)	% in genome	Length (Bp)	% in genome
DNA	38780022	4.640339	3803219	0.455085	68575139	8.205562	86205239	10.31514
LINE	22605365	2.704912	15765928	1.886519	27968712	3.346679	38256371	4.57768
SINE	855850	0.102409	0	0	1335559	0.15981	1846750	0.220978
LTR	10223282	1.223297	3586476	0.42915	5978813	0.715413	15337442	1.835247
Other	8576	0.001026	0	0	0	0	8576	0.001026
Unknown	0	0	0	0	100684493	12.0477	100684493	12.0477
Total	66856408	7.999902	22881095	2.737905	202381509	24.21656	229120570	27.4161

Table S7 Female and male resequencing samples.

sample	sex	read number	length	fold
A2	female	53869319	8080397850	10.49
A3	female	51757835	7763675250	10.08
A4	female	51057957	7658693550	9.95
A5	female	52404374	7860656100	10.21
A6	female	58420260	8763039000	11.38
A7	female	50068514	7510277100	9.75
A9	female	52502798	7875419700	10.23
A10	female	54134204	8120130600	10.55
A11	female	54423132	8163469800	10.60
A12	female	57138574	8570786100	11.13
A15	female	46016314	6902447100	8.96
A16	female	51081572	7662235800	9.95

A17	female	51898796	7784819400	10.11
A18	female	53374918	8006237700	10.40
A19	female	46965060	7044759000	9.15
A20	female	50022580	7503387000	9.74
A21	female	46586012	6987901800	9.08
A22	female	53869319	8080397850	10.49
A23	female	56531672	8479750800	11.01
A24	female	45882578	6882386700	8.94
A25	female	51953546	7793031900	10.12
A26	female	49184938	7377740700	9.58
A28	female	59091284	8863692600	11.51
A29	female	56688662	8503299300	11.04
A30	female	53816930	8072539500	10.48
A31	female	53052986	7957947900	10.33
A33	female	59837208	8975581200	11.66
A34	female	53393038	8008955700	10.40
A35	female	57073794	8561069100	11.12
A36	female	52130834	7819625100	10.16
A37	female	60416664	9062499600	11.77
A38	female	54227870	8134180500	10.56
A41	female	66457486	9968622900	12.95
A43	female	58682292	8802343800	11.43
A44	female	55172230	8275834500	10.75
A46	female	63116090	9467413500	12.30
A47	female	58198228	8729734200	11.34
A48	female	58592070	8788810500	11.41
A49	female	54721274	8208191100	10.66
A50	female	54346646	8151996900	10.59
B6H	female	58083548	8712532200	11.31
B9H	female	57477810	8621671500	11.20
B11	female	60773540	9116031000	11.84
B14	female	61294116	9194117400	11.94
B17	female	54048936	8107340400	10.53
B19	female	50438288	7565743200	9.83
B20	female	47051612	7057741800	9.17
B21	female	48931398	7339709700	9.53
B22	female	50616030	7592404500	9.86
B26	female	51605104	7740765600	10.05
B27	female	57781900	8667285000	11.26
B31	female	46689560	7003434000	9.10
B32	female	55220416	8283062400	10.76
B34	female	58173248	8725987200	11.33
B37	female	54093112	8113966800	10.54
B38	female	53776168	8066425200	10.48

B40	female	50880964	7632144600	9.91
B41	female	57166214	8574932100	11.14
B42	female	56039324	8405898600	10.92
B43	female	59143048	8871457200	11.52
B44	female	46937766	7040664900	9.14
B45	female	54034656	8105198400	10.53
B46	female	51808232	7771234800	10.09
B47	female	50565098	7584764700	9.85
B48	female	52245066	7836759900	10.18
B50	female	64572488	9685873200	12.58
B52	female	57102630	8565394500	11.12
D3	female	59838009	8975701350	11.66
D5	female	55709172	8356375800	10.85
D6	female	51605231	7740784650	10.05
D8	female	47810428	7171564200	9.31
D14	female	55358472	8303770800	10.78
D17	female	63047268	9457090200	12.28
D18	female	50497484	7574622600	9.84
D23	female	54932454	8239868100	10.70
D26	female	55781932	8367289800	10.87
D29	female	55297266	8294589900	10.77
D30	female	51451894	7717784100	10.02
D33	female	49420980	7413147000	9.63
D34	female	55678796	8351819400	10.85
D35	female	65382612	9807391800	12.74
D37	female	49882428	7482364200	9.72
D38	female	53812434	8071865100	10.48
D39	female	51219836	7682975400	9.98
D40	female	47148800	7072320000	9.18
D41	female	55746868	8362030200	10.86
D44	female	53775640	8066346000	10.48
D50	female	57771484	8665722600	11.25
D52	female	55709172	8356375800	10.85
D53	female	52164570	7824685500	10.16
D54	female	58010914	8701637100	11.30
D55	female	47001134	7050170100	9.16
D56	female	56154980	8423247000	10.94
D57	female	69824058	10473608700	13.60
D60	female	50820920	7623138000	9.90
D65	female	48735634	7310345100	9.49
D67	female	54026400	8103960000	10.52
E1	female	55661190	8349178500	10.84
I2	female	60719282	9107892300	11.83
I3	female	54501178	8175176700	10.62

I4	female	49793466	7469019900	9.70
I7	female	62724592	9408688800	12.22
I9	female	51298500	7694775000	9.99
I10	female	51210112	7681516800	9.98
I16	female	62196634	9329495100	12.12
I17	female	56741904	8511285600	11.05
I19	female	58077666	8711649900	11.31
I20	female	54057906	8108685900	10.53
I21	female	59816372	8972455800	11.65
I23	female	58741828	8811274200	11.44
I26	female	60753522	9113028300	11.84
K1	female	56488815	8473322250	11.00
K2	female	58661376	8799206400	11.43
K3	female	52816098	7922414700	10.29
K6	female	60776832	9116524800	11.84
K10	female	50496822	7574523300	9.84
K13	female	52306902	7846035300	10.19
K17	female	63648712	9547306800	12.40
K21	female	54826868	8224030200	10.68
K31	female	56408252	8461237800	10.99
Q2	female	61155452	9173317800	11.91
Q4	female	54060836	8109125400	10.53
Q5	female	59130064	8869509600	11.52
Q8	female	56039316	8405897400	10.92
A1	male	57138574	8570786100	11.13
A8	male	52457870	7868680500	10.22
A13	male	51120782	7668117300	9.96
A14	male	48566986	7285047900	9.46
A27	male	55904512	8385676800	10.89
A32	male	51757835	7763675250	10.08
A39	male	59522826	8928423900	11.60
A40	male	50442758	7566413700	9.83
A42	male	51057957	7658693550	9.95
A45	male	53114560	7967184000	10.35
B1H	male	58022080	8703312000	11.30
B2H	male	52304858	7845728700	10.19
B3H	male	56216602	8432490300	10.95
B4H	male	55647570	8347135500	10.84
B5H	male	52002874	7800431100	10.13
B7H	male	64232632	9634894800	12.51
B12	male	49250576	7387586400	9.59
B13	male	54082312	8112346800	10.54
B15	male	61027136	9154070400	11.89
B16	male	51800316	7770047400	10.09

B18	male	52339818	7850972700	10.20
B23	male	47996578	7199486700	9.35
B24	male	48688578	7303286700	9.48
B25	male	48439138	7265870700	9.44
B28	male	52388524	7858278600	10.21
B29	male	53417174	8012576100	10.41
B30	male	49954024	7493103600	9.73
B33	male	50779242	7616886300	9.89
B35	male	60635140	9095271000	11.81
B36	male	49994244	7499136600	9.74
B39	male	54960490	8244073500	10.71
B49	male	62033488	9305023200	12.08
B51	male	53652150	8047822500	10.45
D1	male	52807373	7921105950	10.29
D9	male	51311460	7696719000	10.00
D12	male	52807373	7921105950	10.29
D15	male	59765892	8964883800	11.64
D16	male	53228364	7984254600	10.37
D27	male	59265376	8889806400	11.55
D31	male	57572606	8635890900	11.22
D32	male	59838009	8975701350	11.66
D36	male	47165304	7074795600	9.19
D43	male	49699262	7454889300	9.68
D45	male	60648522	9097278300	11.81
D48	male	61446742	9217011300	11.97
D58	male	51320112	7698016800	10.00
D59	male	51797712	7769656800	10.09
D63	male	54761352	8214202800	10.67
D68	male	55652972	8347945800	10.84
I1	male	65399993	9809998950	12.74
I5	male	54471664	8170749600	10.61
I6	male	50344492	7551673800	9.81
I8	male	51373112	7705966800	10.01
I11	male	53201248	7980187200	10.36
I12	male	65399993	9809998950	12.74
I13	male	60809162	9121374300	11.85
I14	male	60984010	9147601500	11.88
I15	male	51339038	7700855700	10.00
I18	male	54962918	8244437700	10.71
I22	male	60719282	9107892300	11.83
I24	male	49687804	7453170600	9.68
I25	male	60154978	9023246700	11.72
K4	male	51672858	7750928700	10.07
K5	male	57240454	8586068100	11.15

K7	male	56634770	8495215500	11.03
K8	male	52570050	7885507500	10.24
K9	male	47906880	7186032000	9.33
K11	male	49935176	7490276400	9.73
K12	male	56488815	8473322250	11.00
K26	male	49503934	7425590100	9.64
K27	male	47156518	7073477700	9.19
K30	male	54074580	8111187000	10.53
K32	male	52816098	7922414700	10.29

Table S8 Genes in the inverted region of Y. 24 of 139 genes are named as $Y^{+}X^{-}$-genes and highlighted with red, which are marked with "deleted" using Flo and have no homologous sequence on the corresponding X synteny block.

Gene ID	Nr annotation
Male_chrY_45	Transposable element Tcb1 transposase, partial [Stegodyphus mimosarum]
Male_chrY_70	PREDICTED: uncharacterized protein LOC106675357, partial [Maylandia zebra]
Male_chrY_71	PREDICTED: uncharacterized protein LOC106676632 [Maylandia zebra]
Male_chrY_73	PREDICTED: uncharacterized protein LOC108415997 [Pygocentrus nattereri]
Male_chrY_74	PREDICTED: corepressor interacting with RBPJ 1-like [Pygocentrus nattereri]
Male_chrY_75	PREDICTED: uncharacterized protein LOC106532676, partial [Austrofundulus limnaeus]
Male_chrY_76	hypothetical protein EH28_00122 [Larimichthys crocea]
Male_chrY_92	PREDICTED: bombesin receptor-activated protein C6orf89 homolog [Stegastes partitus]
Male_chrY_104	NA[1]
Male_chrY_107	PREDICTED: uncharacterized protein LOC109078765 [Cyprinus carpio]
Male_chrY_114	PREDICTED: E3 ubiquitin-protein ligase SIAH1-like [Orussus abietinus]
Male_chrY_118	PREDICTED: uncharacterized protein LOC106532676, partial [Austrofundulus limnaeus]
Male_chrY_123	PREDICTED: uncharacterized protein LOC106676632 [Maylandia zebra]
Male_chrY_133	PREDICTED: uncharacterized protein LOC106511041 [Austrofundulus limnaeus]
Male_chrY_134	PREDICTED: uncharacterized protein LOC109060343 [Cyprinus carpio]
Male_chrY_135	NA
Male_chrY_139	PREDICTED: leucine-rich repeats and immunoglobulin-like domains protein 2 [Stegastes partitus]

[1] Not Available, without Nr annotation

Male_chrY_142	NA
Male_chrY_147	hypothetical protein EH28_00905 [Larimichthys crocea]
Male_chrY_149	PREDICTED: uncharacterized protein LOC106511041 [Austrofundulus limnaeus]
Male_chrY_161	PREDICTED: uncharacterized protein LOC106511041 [Austrofundulus limnaeus]
Male_chrY_174	PREDICTED: uncharacterized protein LOC109141790, partial [Larimichthys crocea]
Male_chrY_175	PREDICTED: uncharacterized protein LOC109141461 [Larimichthys crocea]
Male_chrY_184	LIM/homeobox protein Lhx6 [Larimichthys crocea]
Male_chrY_187	hypothetical protein Y1Q_0021212 [Alligator mississippiensis]
Male_chrY_189	PREDICTED: uncharacterized protein LOC105008641 isoform X2 [Esox lucius]
Male_chrY_190	PREDICTED: uncharacterized protein LOC106529025, partial [Austrofundulus limnaeus]
Male_chrY_191	uncharacterized protein LOC109966335 isoform X1 [Monopterus albus]
Male_chrY_201	PREDICTED: G2/M phase-specific E3 ubiquitin-protein ligase [Oreochromis niloticus]
Male_chrY_223	NA
Male_chrY_224	piggyBac transposable element-derived protein 3-like [Labrus bergylta]
Male_chrY_225	PREDICTED: uncharacterized protein LOC106511041 [Austrofundulus limnaeus]
Male_chrY_226	PREDICTED: beta-1,3-galactosyl-O-glycosyl-glycoprotein beta-1,6-N-acetylglucosaminyltransferase 7-like [Lates calcarifer]
Male_chrY_228	PREDICTED: uncharacterized protein LOC108415997 [Pygocentrus nattereri]
Male_chrY_232	NA
Male_chrY_233	PREDICTED: uncharacterized protein LOC106511041 [Austrofundulus limnaeus]
Male_chrY_242	NA
Male_chrY_245	NA
Male_chrY_248	NA
Male_chrY_250	PREDICTED: uncharacterized protein LOC106511041 [Austrofundulus limnaeus]
Male_chrY_251	NA
Male_chrY_253	PREDICTED: uncharacterized protein LOC106906582 isoform X1 [Poecilia mexicana]
Male_chrY_262	PREDICTED: uncharacterized protein LOC107373801 [Nothobranchius furzeri]
Male_chrY_269	PREDICTED: uncharacterized protein LOC106511041 [Austrofundulus limnaeus]
Male_chrY_272	NA
Male_chrY_273	NA
Male_chrY_275	PREDICTED: uncharacterized protein LOC109616568 [Esox lucius]
Male_chrY_276	PREDICTED: putative nuclease HARBI1 [Cyprinus carpio]

Male_chrY_278	uncharacterized protein LOC110004991 [Labrus bergylta]
Male_chrY_279	PREDICTED: uncharacterized protein LOC102194423 [Pundamilia nyererei]
Male_chrY_282	PREDICTED: uncharacterized protein LOC106511041 [Austrofundulus limnaeus]
Male_chrY_288	putative transposase element L1Md-A101/L1Md-A102/L1Md-A2 [Larimichthys crocea]
Male_chrY_293	NA
Male_chrY_302	NA
Male_chrY_304	PREDICTED: uncharacterized protein LOC106532676, partial [Austrofundulus limnaeus]
Male_chrY_308	NA
Male_chrY_316	PREDICTED: uncharacterized protein LOC106520463 [Austrofundulus limnaeus]
Male_chrY_319	NA
Male_chrY_320	NA
Male_chrY_324	uncharacterized protein LOC110959929 [Acanthochromis polyacanthus]
Male_chrY_325	uncharacterized protein LOC110959929 [Acanthochromis polyacanthus]
Male_chrY_326	PREDICTED: uncharacterized protein LOC109084889 [Cyprinus carpio]
Male_chrY_329	NA
Male_chrY_341	NA
Male_chrY_351	PREDICTED: uncharacterized protein LOC102083044 [Oreochromis niloticus]
Male_chrY_369	PREDICTED: uncharacterized protein LOC106157088 [Lingula anatina]
Male_chrY_370	PREDICTED: uncharacterized protein LOC106511041 [Austrofundulus limnaeus]
Male_chrY_405	NA
Male_chrY_408	PREDICTED: uncharacterized protein LOC107372812 isoform X2 [Nothobranchius furzeri]
Male_chrY_432	hypothetical protein EH28_00122 [Larimichthys crocea]
Male_chrY_438	NA
Male_chrY_458	PREDICTED: uncharacterized protein LOC106511041 [Austrofundulus limnaeus]
Male_chrY_480	uncharacterized protein LOC110966885 [Acanthochromis polyacanthus]
Male_chrY_481	PREDICTED: uncharacterized protein LOC106511041 [Austrofundulus limnaeus]
Male_chrY_486	NA
Male_chrY_495	NA
Male_chrY_531	unnamed protein product [Tetraodon nigroviridis]
Male_chrY_532	PREDICTED: uncharacterized protein LOC106520070 [Austrofundulus limnaeus]
Male_chrY_533	PREDICTED: hepatoma-derived growth factor-related protein 2 isoform X1 [Latimeria chalumnae]
Male_chrY_552	PREDICTED: uncharacterized protein LOC106511041 [Austrofundulus limnaeus]

Male_chrY_563	PREDICTED: uncharacterized protein LOC106511041 [Austrofundulus limnaeus]
Male_chrY_588	NA
Male_chrY_595	NA
Male_chrY_630	PREDICTED: uncharacterized protein LOC106532676, partial [Austrofundulus limnaeus]
Male_chrY_631	PREDICTED: uncharacterized protein LOC106511041 [Austrofundulus limnaeus]
Male_chrY_632	PREDICTED: uncharacterized protein LOC106511041 [Austrofundulus limnaeus]
Male_chrY_633	NA
Male_chrY_658	PREDICTED: gastrin-releasing peptide receptor-like [Neolamprologus brichardi]
Male_chrY_677	PREDICTED: uncharacterized protein LOC109060343 [Cyprinus carpio]
Male_chrY_682	NA
Male_chrY_728	NA
Male_chrY_729	NA
Male_chrY_730	PREDICTED: uncharacterized protein LOC106532676, partial [Austrofundulus limnaeus]
Male_chrY_736	NA
Male_chrY_742	PREDICTED: uncharacterized protein LOC106532676, partial [Austrofundulus limnaeus]
Male_chrY_757	NA
Male_chrY_785	NA
Male_chrY_793	PREDICTED: G2/M phase-specific E3 ubiquitin-protein ligase-like [Maylandia zebra]
Male_chrY_797	NA
Male_chrY_798	PREDICTED: uncharacterized protein LOC107098251, partial [Cyprinodon variegatus]
Male_chrY_805	NA
Male_chrY_806	NA
Male_chrY_808	PREDICTED: uncharacterized protein LOC106529025, partial [Austrofundulus limnaeus]
Male_chrY_809	PREDICTED: uncharacterized protein LOC109078765 [Cyprinus carpio]
Male_chrY_837	PREDICTED: P2X purinoceptor 7-like [Nothobranchius furzeri]
Male_chrY_838	PREDICTED: uncharacterized protein LOC106906582 isoform X1 [Poecilia mexicana]
Male_chrY_839	hypothetical protein EH28_00122 [Larimichthys crocea]
Male_chrY_856	PREDICTED: uncharacterized protein LOC106511041 [Austrofundulus limnaeus]
Male_chrY_861	NA
Male_chrY_862	PREDICTED: uncharacterized protein LOC104966217 isoform X1 [Notothenia coriiceps]
Male_chrY_865	PREDICTED: uncharacterized protein LOC106511041 [Austrofundulus

	limnaeus]
Male_chrY_866	PREDICTED: uncharacterized protein LOC109064258 [Cyprinus carpio]
Male_chrY_868	NA
Male_chrY_880	PREDICTED: uncharacterized protein LOC106935616 isoform X3 [Poecilia latipinna]
Male_chrY_885	NA
Male_chrY_893	PREDICTED: hepatoma-derived growth factor-related protein 2 isoform X1 [Latimeria chalumnae]
Male_chrY_894	NA
Male_chrY_895	NA
Male_chrY_897	NA
Male_chrY_898	NA
Male_chrY_899	PREDICTED: uncharacterized protein LOC106532676, partial [Austrofundulus limnaeus]
Male_chrY_900	PREDICTED: uncharacterized protein LOC106511041 [Austrofundulus limnaeus]
Male_chrY_919	PREDICTED: uncharacterized protein LOC108879319, partial [Lates calcarifer]
Male_chrY_923	PREDICTED: uncharacterized protein LOC106511041 [Austrofundulus limnaeus]
Male_chrY_941	PREDICTED: uncharacterized protein LOC106511041 [Austrofundulus limnaeus]
Male_chrY_943	uncharacterized protein LOC110959929 [Acanthochromis polyacanthus]
Male_chrY_955	NA
Male_chrY_961	NA
Male_chrY_991	uncharacterized protein LOC109974095 [Monopterus albus]
Male_chrY_1003	PREDICTED: uncharacterized protein LOC106676632 [Maylandia zebra]
Male_chrY_1006	PREDICTED: chromodomain-helicase-DNA-binding protein 2, partial [Notothenia coriiceps]
Male_chrY_1007	PREDICTED: uncharacterized protein LOC106511041 [Austrofundulus limnaeus]
Male_chrY_1008	NA
Male_chrY_1020	hypothetical protein EH28_00463 [Larimichthys crocea]
Male_chrY_1023	PREDICTED: corepressor interacting with RBPJ 1-like [Pygocentrus nattereri]
Male_chrY_1024	PREDICTED: uncharacterized protein LOC106532676, partial [Austrofundulus limnaeus]
Male_chrY_1027	NA
Male_chrY_1028	PREDICTED: uncharacterized protein LOC106511041 [Austrofundulus limnaeus]
Male_chrY_1030	NA

Table S9 Evolutionary strata in the divergent region of the neo-Y. The divergence

time of sections A, B, C and D is shown with the mean value, while that of section E is shown using a range (48 MYA to near present) to reveal the dynamic and gradual process of divergence evolution.

Evolutionary Strata	Location		Divergence Time
	from (Mb)	to (Mb)	(MYA)
section A	7	18	55
section B	18	21	28
section C	21	31	55
section D	31	41	48
section E	41	54	48~0

Table S10 Eighty-seven DEGs specifically expressed in the testis on the neo-Y based on the transcriptomics analysis of the gonads of the 60th and 80th dph fish.

Gene ID	NR annotation
Male_chrY_62	PX domain-containing protein kinase-like protein isoform X1 [Labrus bergylta]
Male_chrY_65	PREDICTED: uncharacterized protein LOC109646603 [Paralichthys olivaceus]
Male_chrY_79	PREDICTED: coiled-coil domain-containing glutamate-rich protein 1-like [Lates calcarifer]
Male_chrY_82	PREDICTED: retinoblastoma-binding protein 5 [Larimichthys crocea]
Male_chrY_83	Centromere protein P [Larimichthys crocea]
Male_chrY_88	PREDICTED: nucleolar protein 8 [Poecilia mexicana]
Male_chrY_150	SAM pointed domain-containing Ets transcription factor-like isoform X1 [Monopterus albus]
Male_chrY_180	PREDICTED: mitochondrial glutamate carrier 1-like [Lates calcarifer]
Male_chrY_192	protein phosphatase 1H-like [Labrus bergylta]
Male_chrY_194	F-actin-capping protein subunit alpha-1 [Dicentrarchus labrax]
Male_chrY_209	cytochrome c oxidase assembly protein COX14 homolog [Fundulus heteroclitus]
Male_chrY_217	Retinol dehydrogenase 5 [Dicentrarchus labrax]
Male_chrY_249	CTTNBP2 N-terminal-like protein [Dicentrarchus labrax]
Male_chrY_257	PREDICTED: mitochondrial import inner membrane translocase subunit Tim17-A-like [Neolamprologus brichardi]
Male_chrY_260	Importin-9 [Dicentrarchus labrax]
Male_chrY_262	PREDICTED: uncharacterized protein LOC107373801 [Nothobranchius furzeri]

Male_chrY_336	PREDICTED: mRNA turnover protein 4 homolog [Larimichthys crocea]
Male_chrY_342	PREDICTED: protein DJ-1-like [Neolamprologus brichardi]
Male_chrY_349	prothymosin alpha-A-like [Monopterus albus]
Male_chrY_362	PREDICTED: epithelial membrane protein 3-like [Stegastes partitus]
Male_chrY_364	Something about silencing protein 10 [Dicentrarchus labrax]
Male_chrY_377	PREDICTED: twinfilin-2-like [Austrofundulus limnaeus]
Male_chrY_394	Retinoid-binding protein 7 [Larimichthys crocea]
Male_chrY_418	PREDICTED: uncharacterized protein LOC108889887 [Lates calcarifer]
Male_chrY_420	PREDICTED: caveolin-2-like [Stegastes partitus]
Male_chrY_448	PREDICTED: LOW QUALITY PROTEIN: probable tRNA pseudouridine synthase 2 [Lates calcarifer]
Male_chrY_458	PREDICTED: uncharacterized protein LOC106511041 [Austrofundulus limnaeus]
Male_chrY_463	PREDICTED: peptidyl-prolyl cis-trans isomerase-like 1 [Lates calcarifer]
Male_chrY_477	PREDICTED: aminoacylase-1 [Lates calcarifer]
Male_chrY_517	PREDICTED: transmembrane protein 82-like [Paralichthys olivaceus]
Male_chrY_527	PREDICTED: RNA polymerase II subunit A C-terminal domain phosphatase SSU72 [Larimichthys crocea]
Male_chrY_537	PREDICTED: dual serine/threonine and tyrosine protein kinase [Paralichthys olivaceus]
Male_chrY_545	PREDICTED: serine/arginine-rich splicing factor 3 isoform X1 [Paralichthys olivaceus]
Male_chrY_557	PREDICTED: interferon regulatory factor 6 [Paralichthys olivaceus]
Male_chrY_592	PREDICTED: actin-related protein 2/3 complex subunit 4 [Ficedula albicollis]
Male_chrY_598	PREDICTED: ruvB-like 1 [Lates calcarifer]
Male_chrY_608	PREDICTED: von Hippel-Lindau disease tumor suppressor [Stegastes partitus]
Male_chrY_610	PREDICTED: protein cereblon [Maylandia zebra]
Male_chrY_616	peptidyl-prolyl cis-trans isomerase FKBP11 [Labrus bergylta]
Male_chrY_654	class E basic helix-loop-helix protein 40-like [Acanthochromis polyacanthus]
Male_chrY_659	PREDICTED: sorting nexin-6-like [Larimichthys crocea]
Male_chrY_680	PREDICTED: cyclin-dependent kinase 4 [Lates calcarifer]
Male_chrY_697	PREDICTED: uncharacterized protein C1orf106-like isoform X1 [Larimichthys crocea]
Male_chrY_716	PREDICTED: bile acid-CoA:amino acid N-acyltransferase-like isoform X3 [Lates calcarifer]
Male_chrY_718	PREDICTED: translocon-associated protein subunit alpha-like [Stegastes partitus]
Male_chrY_744	myogenin [Siniperca chuatsi]
Male_chrY_792	Keratin, type II cytoskeletal 8 [Dicentrarchus labrax]
Male_chrY_804	MICOS complex subunit mic25a-like isoform X2 [Acanthochromis polyacanthus]

Male_chrY_845	PREDICTED: promotilin-like [Stegastes partitus]
Male_chrY_850	PREDICTED: bis(5'-nucleosyl)-tetraphosphatase [asymmetrical] isoform X1 [Larimichthys crocea]
Male_chrY_857	PREDICTED: calpastatin isoform X11 [Lates calcarifer]
Male_chrY_871	Cell growth-regulating nucleolar protein [Dicentrarchus labrax]
Male_chrY_929	PREDICTED: RNA-binding protein 38-like isoform X1 [Lates calcarifer]
Male_chrY_930	mRNA export factor [Dicentrarchus labrax]
Male_chrY_934	PREDICTED: dysbindin-like [Lates calcarifer]
Male_chrY_936	PREDICTED: LOW QUALITY PROTEIN: cadherin-like protein 26 [Lates calcarifer]
Male_chrY_952	DNA-binding protein inhibitor ID-1 isoform X2 [Boleophthalmus pectinirostris]
Male_chrY_957	PHD finger protein 20 [Dicentrarchus labrax]
Male_chrY_965	PREDICTED: DNA topoisomerase 1 [Paralichthys olivaceus]
Male_chrY_968	Protein FAM83D [Dicentrarchus labrax]
Male_chrY_973	GATA-binding factor 3 [Dicentrarchus labrax]
Male_chrY_982	PREDICTED: CXXC-type zinc finger protein 1 [Larimichthys crocea]
Male_chrY_984	PREDICTED: coiled-coil domain-containing protein 120 [Larimichthys crocea]
Male_chrY_1000	thioredoxin reductase 3 [Oplegnathus fasciatus]
Male_chrY_1048	PREDICTED: PRA1 family protein 3-like [Paralichthys olivaceus]
Male_chrY_1049	tRNA-nucleotidyltransferase 1, mitochondrial [Dicentrarchus labrax]
Male_chrY_1067	Peptidyl-tRNA hydrolase, putative [Dicentrarchus labrax]
Male_chrY_1078	PREDICTED: casein kinase II subunit alpha-like [Stegastes partitus]
Male_chrY_1099	PREDICTED: zinc finger and SCAN domain-containing protein 32-like [Lates calcarifer]
Male_chrY_1100	PREDICTED: LOW QUALITY PROTEIN: zinc finger CCCH domain-containing protein 11A-like [Lates calcarifer]
Male_chrY_1147	cell division control protein 42 homolog isoform 1 [Danio rerio]
Male_chrY_1173	PREDICTED: nuclear ubiquitous casein and cyclin-dependent kinase substrate 1 [Paralichthys olivaceus]
Male_chrY_1185	PREDICTED: kinetochore protein Nuf2 [Larimichthys crocea]
Male_chrY_1225	PREDICTED: C->U-editing enzyme APOBEC-2-like [Larimichthys crocea]
Male_chrY_1360	PREDICTED: uncharacterized protein LOC108902242 [Lates calcarifer]
Male_chrY_1377	PREDICTED: LOW QUALITY PROTEIN: Fanconi anemia group A protein [Lates calcarifer]
Male_chrY_1379	PREDICTED: charged multivesicular body protein 1a [Maylandia zebra]
Male_chrY_1420	PREDICTED: protein preY, mitochondrial [Lates calcarifer]
Male_chrY_1424	PREDICTED: BTB/POZ domain-containing protein 10-like isoform X2 [Lates calcarifer]
Male_chrY_1457	group XV phospholipase A2 [Sparus aurata]
Male_chrY_1484	PREDICTED: alpha-parvin isoform X1 [Larimichthys crocea]

Male_chrY_1490	PREDICTED: anaphase-promoting complex subunit 13 [Oreochromis niloticus]
Male_chrY_1517	N-lysine methyltransferase SETD6 [Monopterus albus]
Male_chrY_1578	bcl-2-like protein 13 [Monopterus albus]
Male_chrY_1607	PREDICTED: transmembrane protein 5 [Lates calcarifer]
Male_chrY_1627	PREDICTED: electron transfer flavoprotein beta subunit lysine methyltransferase isoform X1 [Larimichthys crocea]
Male_chrY_1770	PREDICTED: dynein light chain roadblock-type 2 [Takifugu rubripes]

Table S11 Statistics of sequencing data for female and male genome assembly.

Type	Female		Male	
	Raw data (bp)	Depth (X)	Raw data (bp)	Depth (X)
10X	123649035600	161	153555804600	201
PacBio	-	-	130311241167	170
Nanopore	-	-	73201944302	96
Hi-C	121037696800	158	148751230000	195

Table S12 Primers for PCR validation of male-specific sequences.

	Gene	Primer Name	Sequence (5'-3')
cDNA	Male_chrY_934	934L	CAAGTTGAGAGAGAGACA
		934R	AGGTGGAGATCTGACTAC
	Male_chrY_1067	1067L	CCATGTACTACTTCAACAAG
		1067R	AGTAGATGAGCCACATTG

Table S13 Accession numbers of the sequencing data and assemblies in the CNGB Nucleotide Sequence Archive database under Bioproject accession CNP0001488.

Sample	Description	Platform	Experiment or Assembly Accession	Run Accession
OpuF_chrv1	female assembly	-	CNA0019300	-
OpuM_chrv1	male assembly	-	CNA0019299	-
F_G_10X	female raw data	BGISEQ-500	CNX0291024	CNR0360831
F_G_HiC	female raw data	BGISEQ-500	CNX0291025	CNR0360832
M_G_NP	male raw data	PromethION	CNX0291026	CNR0360833
M_G_PB	male raw data	PacBio RS II	CNX0291027	CNR0360834
M_G_10X	male raw data	BGISEQ-500	CNX0291028	CNR0360835
M_G_HiC	male raw data	BGISEQ-500	CNX0291029	CNR0360836

F_Trans60_Dup1	transcriptome	BGISEQ-500	CNX0291030	CNR0360837
F_Trans60_Dup2	transcriptome	BGISEQ-500	CNX0291031	CNR0360838
F_Trans60_Dup3	transcriptome	BGISEQ-500	CNX0291032	CNR0360839
F_Trans80_Dup1	transcriptome	BGISEQ-500	CNX0291033	CNR0360840
F_Trans80_Dup2	transcriptome	BGISEQ-500	CNX0291034	CNR0360841
F_Trans80_Dup3	transcriptome	BGISEQ-500	CNX0291035	CNR0360842
M_Trans60_Dup1	transcriptome	BGISEQ-500	CNX0291036	CNR0360843
M_Trans60_Dup2	transcriptome	BGISEQ-500	CNX0291037	CNR0360844
M_Trans60_Dup3	transcriptome	BGISEQ-500	CNX0291038	CNR0360845
M_Trans80_Dup1	transcriptome	BGISEQ-500	CNX0291039	CNR0360846
M_Trans80_Dup2	transcriptome	BGISEQ-500	CNX0291040	CNR0360847
M_Trans80_Dup3	transcriptome	BGISEQ-500	CNX0291041	CNR0360848
Reseq_A1	resequencing	HiSeq X Ten	CNX0291042	CNR0360849
Reseq_A2	resequencing	HiSeq X Ten	CNX0291043	CNR0360850
Reseq_A3	resequencing	HiSeq X Ten	CNX0291044	CNR0360851
Reseq_A4	resequencing	HiSeq X Ten	CNX0291045	CNR0360852
Reseq_A5	resequencing	HiSeq X Ten	CNX0291046	CNR0360853
Reseq_A6	resequencing	HiSeq X Ten	CNX0291047	CNR0360854
Reseq_A7	resequencing	HiSeq X Ten	CNX0291048	CNR0360855
Reseq_A8	resequencing	HiSeq X Ten	CNX0291049	CNR0360856
Reseq_A9	resequencing	HiSeq X Ten	CNX0291050	CNR0360857
Reseq_A10	resequencing	HiSeq X Ten	CNX0291051	CNR0360858
Reseq_A11	resequencing	HiSeq X Ten	CNX0291052	CNR0360859
Reseq_A12	resequencing	HiSeq X Ten	CNX0291053	CNR0360860
Reseq_A13	resequencing	HiSeq X Ten	CNX0291054	CNR0360861
Reseq_A14	resequencing	HiSeq X Ten	CNX0291055	CNR0360862
Reseq_A15	resequencing	HiSeq X Ten	CNX0291056	CNR0360863
Reseq_A16	resequencing	HiSeq X Ten	CNX0291057	CNR0360864
Reseq_A17	resequencing	HiSeq X Ten	CNX0291058	CNR0360865
Reseq_A18	resequencing	HiSeq X Ten	CNX0291059	CNR0360866
Reseq_A19	resequencing	HiSeq X Ten	CNX0291060	CNR0360867
Reseq_A20	resequencing	HiSeq X Ten	CNX0291061	CNR0360868
Reseq_A21	resequencing	HiSeq X Ten	CNX0291062	CNR0360869
Reseq_A22	resequencing	HiSeq X Ten	CNX0291063	CNR0360870
Reseq_A23	resequencing	HiSeq X Ten	CNX0291064	CNR0360871
Reseq_A24	resequencing	HiSeq X Ten	CNX0291065	CNR0360872
Reseq_A25	resequencing	HiSeq X Ten	CNX0291066	CNR0360873
Reseq_A26	resequencing	HiSeq X Ten	CNX0291067	CNR0360874
Reseq_A27	resequencing	HiSeq X Ten	CNX0291068	CNR0360875
Reseq_A28	resequencing	HiSeq X Ten	CNX0291069	CNR0360876
Reseq_A29	resequencing	HiSeq X Ten	CNX0291070	CNR0360877
Reseq_A30	resequencing	HiSeq X Ten	CNX0291071	CNR0360878
Reseq_A31	resequencing	HiSeq X Ten	CNX0291072	CNR0360879
Reseq_A32	resequencing	HiSeq X Ten	CNX0291073	CNR0360880

Reseq_A33	resequencing	HiSeq X Ten	CNX0291074	CNR0360881
Reseq_A34	resequencing	HiSeq X Ten	CNX0291075	CNR0360882
Reseq_A35	resequencing	HiSeq X Ten	CNX0291076	CNR0360883
Reseq_A36	resequencing	HiSeq X Ten	CNX0291077	CNR0360884
Reseq_A37	resequencing	HiSeq X Ten	CNX0291078	CNR0360885
Reseq_A38	resequencing	HiSeq X Ten	CNX0291079	CNR0360886
Reseq_A39	resequencing	HiSeq X Ten	CNX0291080	CNR0360887
Reseq_A40	resequencing	HiSeq X Ten	CNX0291081	CNR0360888
Reseq_A41	resequencing	HiSeq X Ten	CNX0291082	CNR0360889
Reseq_A42	resequencing	HiSeq X Ten	CNX0291083	CNR0360890
Reseq_A43	resequencing	HiSeq X Ten	CNX0291084	CNR0360891
Reseq_A44	resequencing	HiSeq X Ten	CNX0291085	CNR0360892
Reseq_A45	resequencing	HiSeq X Ten	CNX0291086	CNR0360893
Reseq_A46	resequencing	HiSeq X Ten	CNX0291087	CNR0360894
Reseq_A47	resequencing	HiSeq X Ten	CNX0291088	CNR0360895
Reseq_A48	resequencing	HiSeq X Ten	CNX0291089	CNR0360896
Reseq_A49	resequencing	HiSeq X Ten	CNX0291090	CNR0360897
Reseq_A50	resequencing	HiSeq X Ten	CNX0291091	CNR0360898
Reseq_B1H	resequencing	HiSeq X Ten	CNX0291092	CNR0360899
Reseq_B2H	resequencing	HiSeq X Ten	CNX0291093	CNR0360900
Reseq_B3H	resequencing	HiSeq X Ten	CNX0291094	CNR0360901
Reseq_B4H	resequencing	HiSeq X Ten	CNX0291095	CNR0360902
Reseq_B5H	resequencing	HiSeq X Ten	CNX0291096	CNR0360903
Reseq_B6H	resequencing	HiSeq X Ten	CNX0291097	CNR0360904
Reseq_B7H	resequencing	HiSeq X Ten	CNX0291098	CNR0360905
Reseq_B9H	resequencing	HiSeq X Ten	CNX0291099	CNR0360906
Reseq_B11	resequencing	HiSeq X Ten	CNX0291100	CNR0360907
Reseq_B12	resequencing	HiSeq X Ten	CNX0291101	CNR0360908
Reseq_B13	resequencing	HiSeq X Ten	CNX0291102	CNR0360909
Reseq_B14	resequencing	HiSeq X Ten	CNX0291103	CNR0360910
Reseq_B15	resequencing	HiSeq X Ten	CNX0291104	CNR0360911
Reseq_B16	resequencing	HiSeq X Ten	CNX0291105	CNR0360912
Reseq_B17	resequencing	HiSeq X Ten	CNX0291106	CNR0360913
Reseq_B18	resequencing	HiSeq X Ten	CNX0291107	CNR0360914
Reseq_B19	resequencing	HiSeq X Ten	CNX0291108	CNR0360915
Reseq_B20	resequencing	HiSeq X Ten	CNX0291109	CNR0360916
Reseq_B21	resequencing	HiSeq X Ten	CNX0291110	CNR0360917
Reseq_B22	resequencing	HiSeq X Ten	CNX0291111	CNR0360918
Reseq_B23	resequencing	HiSeq X Ten	CNX0291112	CNR0360919
Reseq_B24	resequencing	HiSeq X Ten	CNX0291113	CNR0360920
Reseq_B25	resequencing	HiSeq X Ten	CNX0291114	CNR0360921
Reseq_B26	resequencing	HiSeq X Ten	CNX0291115	CNR0360922
Reseq_B27	resequencing	HiSeq X Ten	CNX0291116	CNR0360923
Reseq_B28	resequencing	HiSeq X Ten	CNX0291117	CNR0360924

Reseq_B29	resequencing	HiSeq X Ten	CNX0291118	CNR0360925
Reseq_B30	resequencing	HiSeq X Ten	CNX0291119	CNR0360926
Reseq_B31	resequencing	HiSeq X Ten	CNX0291120	CNR0360927
Reseq_B32	resequencing	HiSeq X Ten	CNX0291121	CNR0360928
Reseq_B33	resequencing	HiSeq X Ten	CNX0291122	CNR0360929
Reseq_B34	resequencing	HiSeq X Ten	CNX0291123	CNR0360930
Reseq_B35	resequencing	HiSeq X Ten	CNX0291124	CNR0360931
Reseq_B36	resequencing	HiSeq X Ten	CNX0291125	CNR0360932
Reseq_B37	resequencing	HiSeq X Ten	CNX0291126	CNR0360933
Reseq_B38	resequencing	HiSeq X Ten	CNX0291127	CNR0360934
Reseq_B39	resequencing	HiSeq X Ten	CNX0291128	CNR0360935
Reseq_B40	resequencing	HiSeq X Ten	CNX0291129	CNR0360936
Reseq_B41	resequencing	HiSeq X Ten	CNX0291130	CNR0360937
Reseq_B42	resequencing	HiSeq X Ten	CNX0291131	CNR0360938
Reseq_B43	resequencing	HiSeq X Ten	CNX0291132	CNR0360939
Reseq_B44	resequencing	HiSeq X Ten	CNX0291133	CNR0360940
Reseq_B45	resequencing	HiSeq X Ten	CNX0291134	CNR0360941
Reseq_B46	resequencing	HiSeq X Ten	CNX0291135	CNR0360942
Reseq_B47	resequencing	HiSeq X Ten	CNX0291136	CNR0360943
Reseq_B48	resequencing	HiSeq X Ten	CNX0291137	CNR0360944
Reseq_B49	resequencing	HiSeq X Ten	CNX0291138	CNR0360945
Reseq_B50	resequencing	HiSeq X Ten	CNX0291139	CNR0360946
Reseq_B51	resequencing	HiSeq X Ten	CNX0291140	CNR0360947
Reseq_B52	resequencing	HiSeq X Ten	CNX0291141	CNR0360948
Reseq_D1	resequencing	HiSeq X Ten	CNX0291142	CNR0360949
Reseq_D3	resequencing	HiSeq X Ten	CNX0291143	CNR0360950
Reseq_D5	resequencing	HiSeq X Ten	CNX0291144	CNR0360951
Reseq_D6	resequencing	HiSeq X Ten	CNX0291145	CNR0360952
Reseq_D8	resequencing	HiSeq X Ten	CNX0291146	CNR0360953
Reseq_D9	resequencing	HiSeq X Ten	CNX0291147	CNR0360954
Reseq_D12	resequencing	HiSeq X Ten	CNX0291148	CNR0360955
Reseq_D14	resequencing	HiSeq X Ten	CNX0291149	CNR0360956
Reseq_D15	resequencing	HiSeq X Ten	CNX0291150	CNR0360957
Reseq_D16	resequencing	HiSeq X Ten	CNX0291151	CNR0360958
Reseq_D17	resequencing	HiSeq X Ten	CNX0291152	CNR0360959
Reseq_D18	resequencing	HiSeq X Ten	CNX0291153	CNR0360960
Reseq_D23	resequencing	HiSeq X Ten	CNX0291154	CNR0360961
Reseq_D26	resequencing	HiSeq X Ten	CNX0291155	CNR0360962
Reseq_D27	resequencing	HiSeq X Ten	CNX0291156	CNR0360963
Reseq_D29	resequencing	HiSeq X Ten	CNX0291157	CNR0360964
Reseq_D30	resequencing	HiSeq X Ten	CNX0291158	CNR0360965
Reseq_D31	resequencing	HiSeq X Ten	CNX0291159	CNR0360966
Reseq_D32	resequencing	HiSeq X Ten	CNX0291160	CNR0360967
Reseq_D33	resequencing	HiSeq X Ten	CNX0291161	CNR0360968

Reseq_D34	resequencing	HiSeq X Ten	CNX0291162	CNR0360969
Reseq_D35	resequencing	HiSeq X Ten	CNX0291163	CNR0360970
Reseq_D36	resequencing	HiSeq X Ten	CNX0291164	CNR0360971
Reseq_D37	resequencing	HiSeq X Ten	CNX0291165	CNR0360972
Reseq_D38	resequencing	HiSeq X Ten	CNX0291166	CNR0360973
Reseq_D39	resequencing	HiSeq X Ten	CNX0291167	CNR0360974
Reseq_D40	resequencing	HiSeq X Ten	CNX0291168	CNR0360975
Reseq_D41	resequencing	HiSeq X Ten	CNX0291169	CNR0360976
Reseq_D43	resequencing	HiSeq X Ten	CNX0291170	CNR0360977
Reseq_D44	resequencing	HiSeq X Ten	CNX0291171	CNR0360978
Reseq_D45	resequencing	HiSeq X Ten	CNX0291172	CNR0360979
Reseq_D48	resequencing	HiSeq X Ten	CNX0291173	CNR0360980
Reseq_D50	resequencing	HiSeq X Ten	CNX0291174	CNR0360981
Reseq_D52	resequencing	HiSeq X Ten	CNX0291175	CNR0360982
Reseq_D53	resequencing	HiSeq X Ten	CNX0291176	CNR0360983
Reseq_D54	resequencing	HiSeq X Ten	CNX0291177	CNR0360984
Reseq_D55	resequencing	HiSeq X Ten	CNX0291178	CNR0360985
Reseq_D56	resequencing	HiSeq X Ten	CNX0291179	CNR0360986
Reseq_D57	resequencing	HiSeq X Ten	CNX0291180	CNR0360987
Reseq_D58	resequencing	HiSeq X Ten	CNX0291181	CNR0360988
Reseq_D59	resequencing	HiSeq X Ten	CNX0291182	CNR0360989
Reseq_D60	resequencing	HiSeq X Ten	CNX0291183	CNR0360990
Reseq_D63	resequencing	HiSeq X Ten	CNX0291184	CNR0360991
Reseq_D65	resequencing	HiSeq X Ten	CNX0291185	CNR0360992
Reseq_D67	resequencing	HiSeq X Ten	CNX0291186	CNR0360993
Reseq_D68	resequencing	HiSeq X Ten	CNX0291187	CNR0360994
Reseq_E1	resequencing	HiSeq X Ten	CNX0291188	CNR0360995
Reseq_I1	resequencing	HiSeq X Ten	CNX0291189	CNR0360996
Reseq_I2	resequencing	HiSeq X Ten	CNX0291190	CNR0360997
Reseq_I3	resequencing	HiSeq X Ten	CNX0291191	CNR0360998
Reseq_I4	resequencing	HiSeq X Ten	CNX0291192	CNR0360999
Reseq_I5	resequencing	HiSeq X Ten	CNX0291193	CNR0361000
Reseq_I6	resequencing	HiSeq X Ten	CNX0291194	CNR0361001
Reseq_I7	resequencing	HiSeq X Ten	CNX0291195	CNR0361002
Reseq_I8	resequencing	HiSeq X Ten	CNX0291196	CNR0361003
Reseq_I9	resequencing	HiSeq X Ten	CNX0291197	CNR0361004
Reseq_I10	resequencing	HiSeq X Ten	CNX0291198	CNR0361005
Reseq_I11	resequencing	HiSeq X Ten	CNX0291199	CNR0361006
Reseq_I12	resequencing	HiSeq X Ten	CNX0291200	CNR0361007
Reseq_I13	resequencing	HiSeq X Ten	CNX0291201	CNR0361008
Reseq_I14	resequencing	HiSeq X Ten	CNX0291202	CNR0361009
Reseq_I15	resequencing	HiSeq X Ten	CNX0291203	CNR0361010
Reseq_I16	resequencing	HiSeq X Ten	CNX0291204	CNR0361011
Reseq_I17	resequencing	HiSeq X Ten	CNX0291205	CNR0361012

Reseq_I18	resequencing	HiSeq X Ten	CNX0291206	CNR0361013
Reseq_I19	resequencing	HiSeq X Ten	CNX0291207	CNR0361014
Reseq_I20	resequencing	HiSeq X Ten	CNX0291208	CNR0361015
Reseq_I21	resequencing	HiSeq X Ten	CNX0291209	CNR0361016
Reseq_I22	resequencing	HiSeq X Ten	CNX0291210	CNR0361017
Reseq_I23	resequencing	HiSeq X Ten	CNX0291211	CNR0361018
Reseq_I24	resequencing	HiSeq X Ten	CNX0291212	CNR0361019
Reseq_I25	resequencing	HiSeq X Ten	CNX0291213	CNR0361020
Reseq_I26	resequencing	HiSeq X Ten	CNX0291214	CNR0361021
Reseq_K1	resequencing	HiSeq X Ten	CNX0291215	CNR0361022
Reseq_K2	resequencing	HiSeq X Ten	CNX0291216	CNR0361023
Reseq_K3	resequencing	HiSeq X Ten	CNX0291217	CNR0361024
Reseq_K4	resequencing	HiSeq X Ten	CNX0291218	CNR0361025
Reseq_K5	resequencing	HiSeq X Ten	CNX0291219	CNR0361026
Reseq_K6	resequencing	HiSeq X Ten	CNX0291220	CNR0361027
Reseq_K7	resequencing	HiSeq X Ten	CNX0291221	CNR0361028
Reseq_K8	resequencing	HiSeq X Ten	CNX0291222	CNR0361029
Reseq_K9	resequencing	HiSeq X Ten	CNX0291223	CNR0361030
Reseq_K10	resequencing	HiSeq X Ten	CNX0291224	CNR0361031
Reseq_K11	resequencing	HiSeq X Ten	CNX0291225	CNR0361032
Reseq_K12	resequencing	HiSeq X Ten	CNX0291226	CNR0361033
Reseq_K13	resequencing	HiSeq X Ten	CNX0291227	CNR0361034
Reseq_K17	resequencing	HiSeq X Ten	CNX0291228	CNR0361035
Reseq_K21	resequencing	HiSeq X Ten	CNX0291229	CNR0361036
Reseq_K26	resequencing	HiSeq X Ten	CNX0291230	CNR0361037
Reseq_K27	resequencing	HiSeq X Ten	CNX0291231	CNR0361038
Reseq_K30	resequencing	HiSeq X Ten	CNX0291232	CNR0361039
Reseq_K31	resequencing	HiSeq X Ten	CNX0291233	CNR0361040
Reseq_K32	resequencing	HiSeq X Ten	CNX0291234	CNR0361041
Reseq_Q2	resequencing	HiSeq X Ten	CNX0291235	CNR0361042
Reseq_Q4	resequencing	HiSeq X Ten	CNX0291236	CNR0361043
Reseq_Q5	resequencing	HiSeq X Ten	CNX0291237	CNR0361044
Reseq_Q8	resequencing	HiSeq X Ten	CNX0291238	CNR0361045

Chen S, Zhang G, Shao C, Huang Q, Liu G, Zhang P, Song W, An N, Chalopin D, Volff JN, et al. 2014. Whole-genome sequence of a flatfish provides insights into ZW sex chromosome evolution and adaptation to a benthic lifestyle. Nat Genet 46:253-260.

Li PZ, Cao DD, Liu XB, Wang YJ, Yu HY, Li XJ, Zhang QQ, Wang XB. 2016. Karyotype analysis and ribosomal gene localization of spotted knifejaw Oplegnathus punctatus. Genet Mol Res 15.

Tang Y, Liu X, Wang J, Li M, Wang Q, Tian F, Su Z, Pan Y, Liu D, Lipka AE, et al. 2016. GAPIT Version 2: An enhanced integrated tool for genomic association and prediction. Plant Genome 9.
