# Supplementary figures and images for: Reconstruction of the Origin of a Neo-Y Sex Chromosome and Its Evolution in the Spotted Knifejaw, Oplegnathus punctatus
Source: Mol Biol Evol. 2021 Mar 9;38(6):2615–26. doi: 10.1093/molbev/msab056 (PMC8136494; doi:10.1093/molbev/msab056)

a

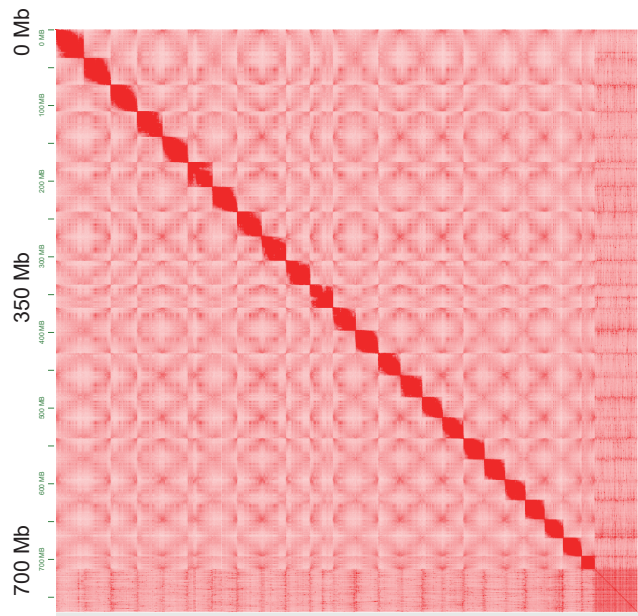

b

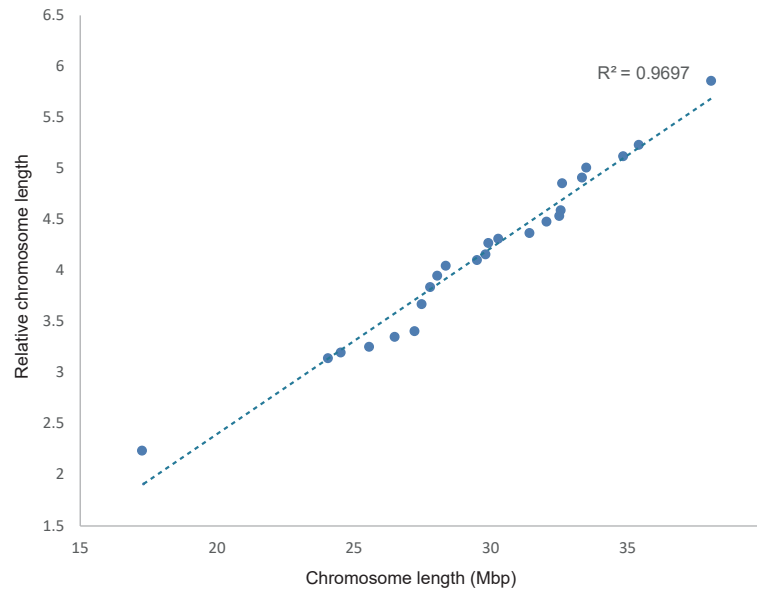

Supplement: msab056_Supplementary_Data [file msab056_supplementary_data.zip › Supplementary Fig. S1.pdf]

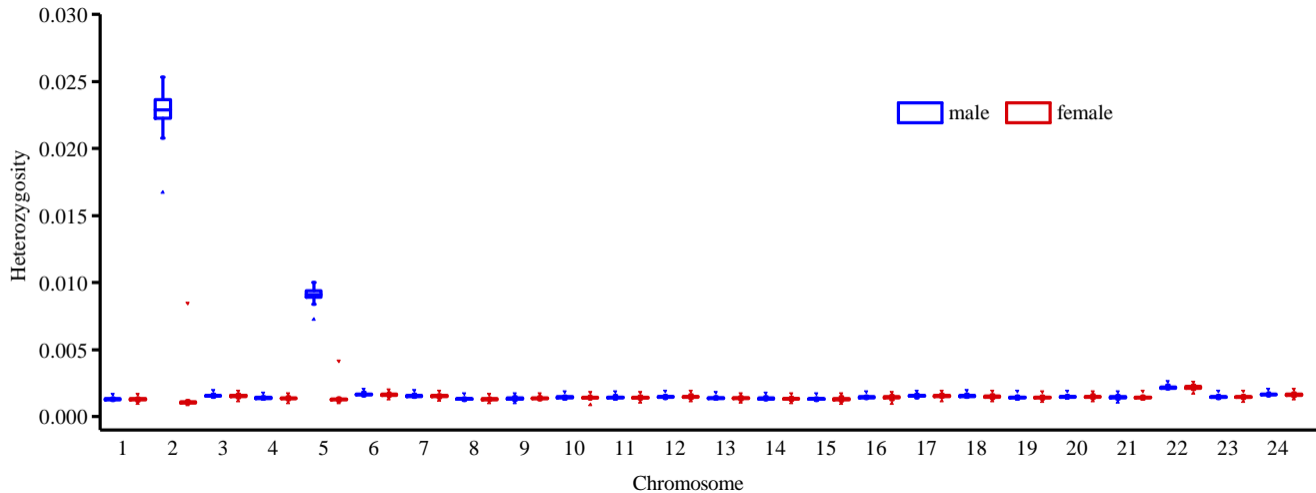

Supplement: msab056_Supplementary_Data [file msab056_supplementary_data.zip › Supplementary Fig. S10.pdf]

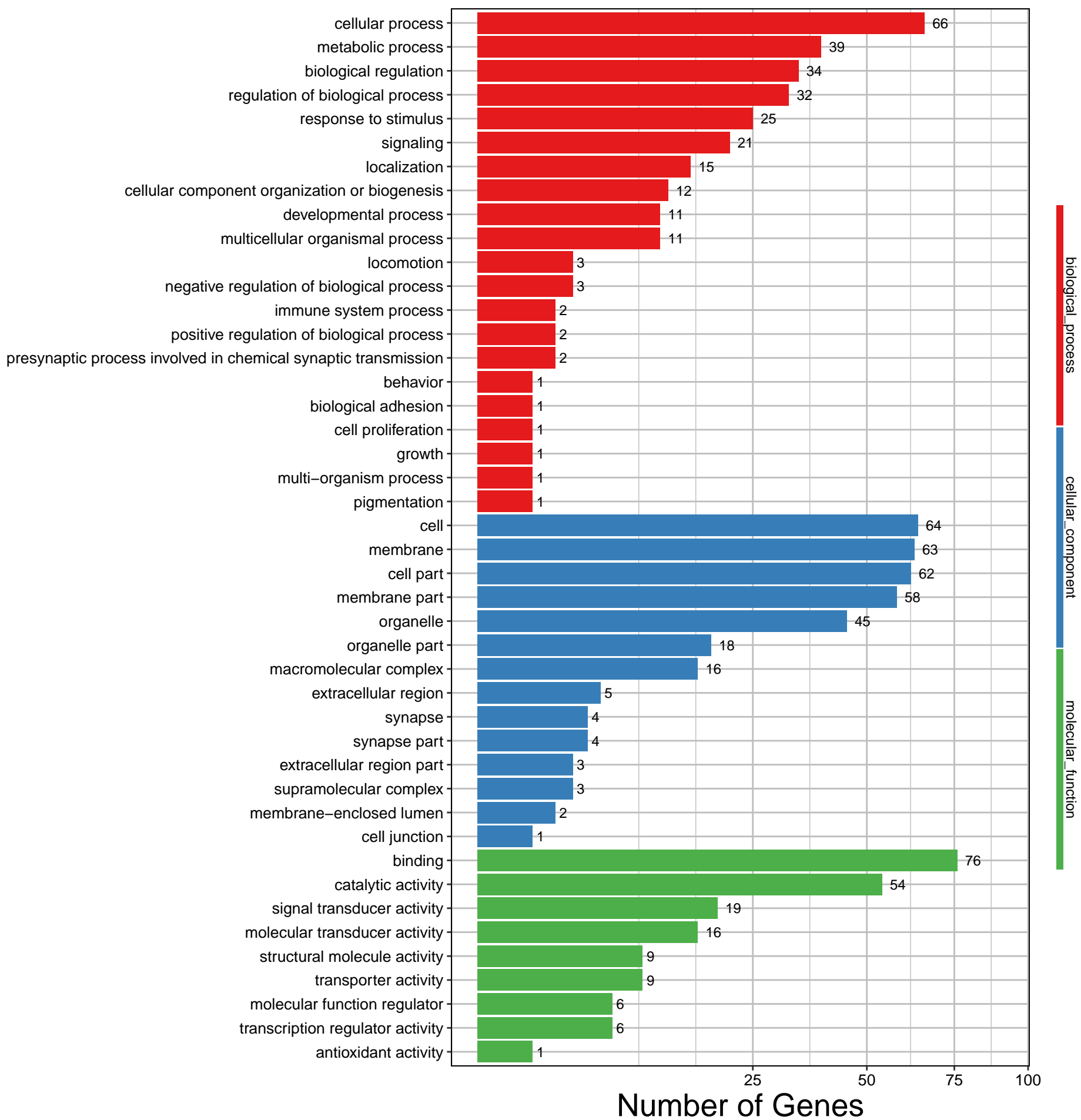

Supplement: msab056_Supplementary_Data [file msab056_supplementary_data.zip › Supplementary Fig. S11.pdf]

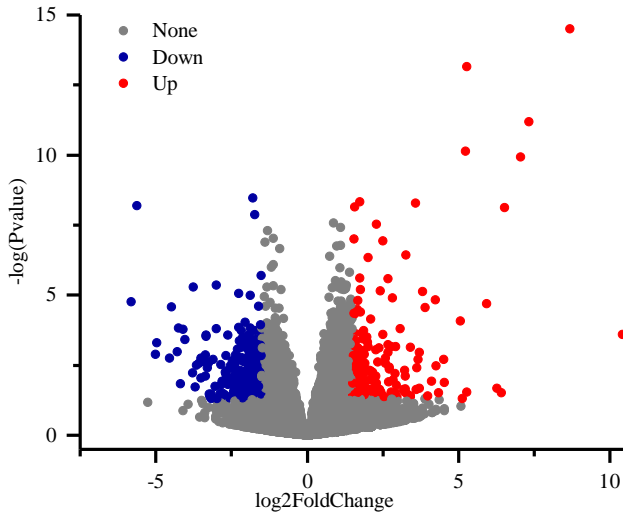

Supplement: msab056_Supplementary_Data [file msab056_supplementary_data.zip › Supplementary Fig. S12.pdf]

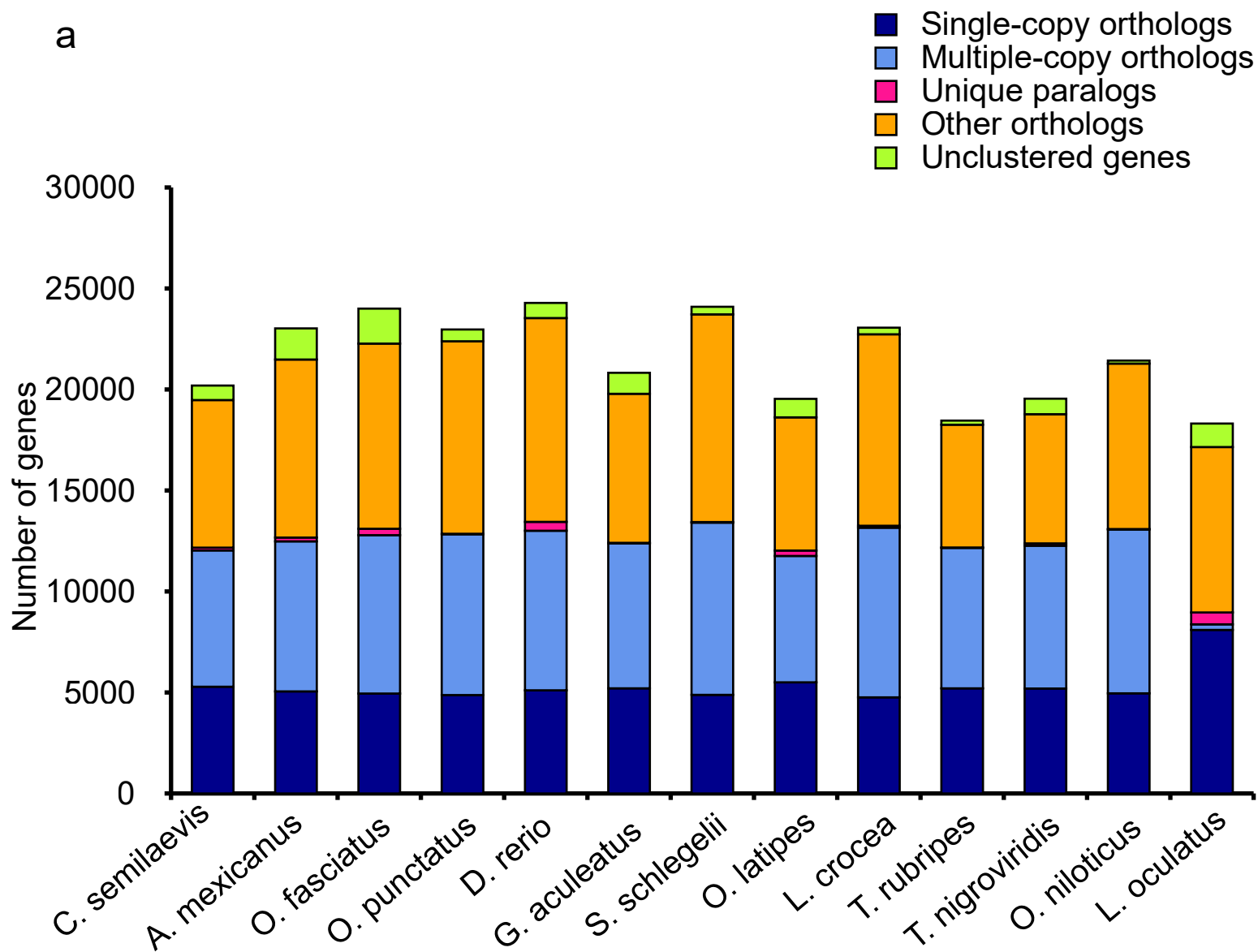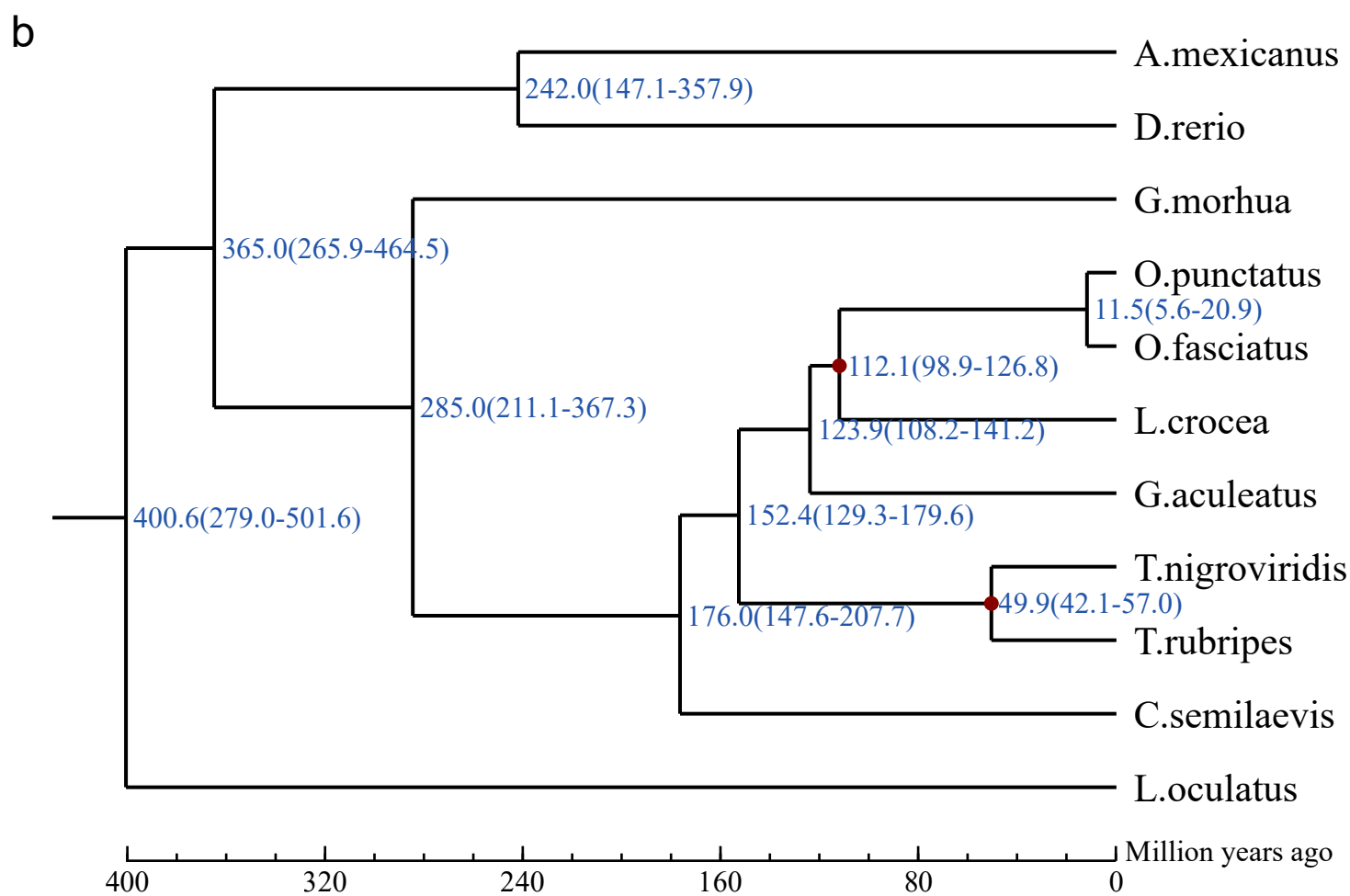

Supplement: msab056_Supplementary_Data [file msab056_supplementary_data.zip › Supplementary Fig. S14.pdf]

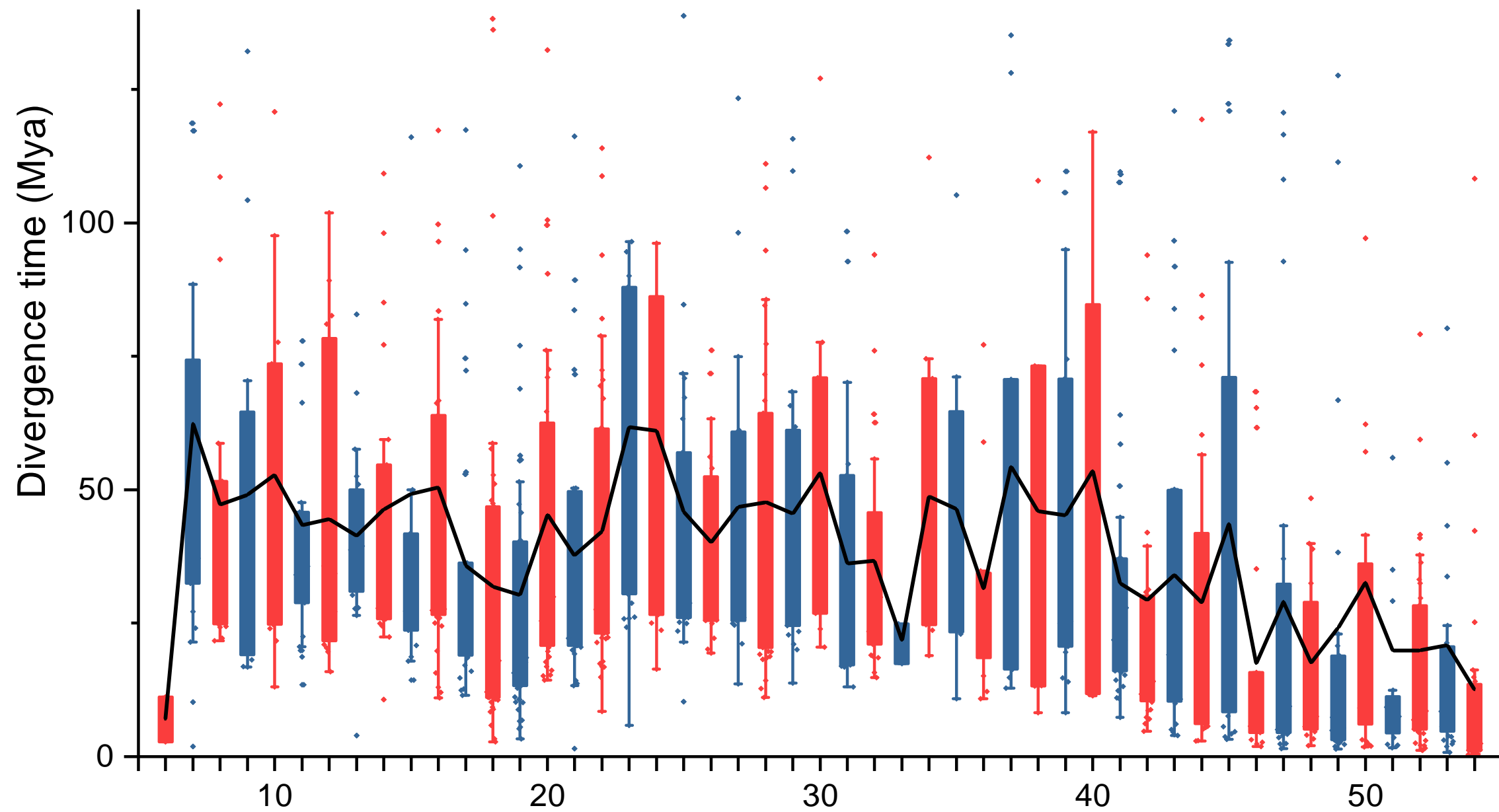

Supplement: msab056_Supplementary_Data [file msab056_supplementary_data.zip › Supplementary Fig. S15.pdf]

Ovary

Testis

M

a

Ovary

Testis

M

b

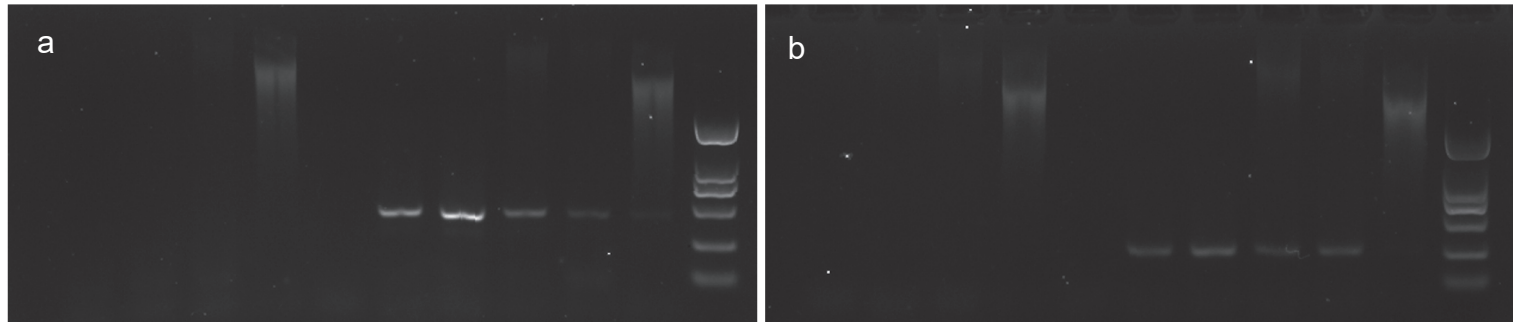

Supplement: msab056_Supplementary_Data [file msab056_supplementary_data.zip › Supplementary Fig. S16 cDNA_RNA.pdf]

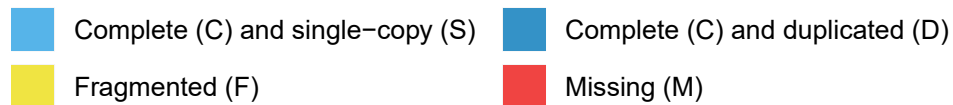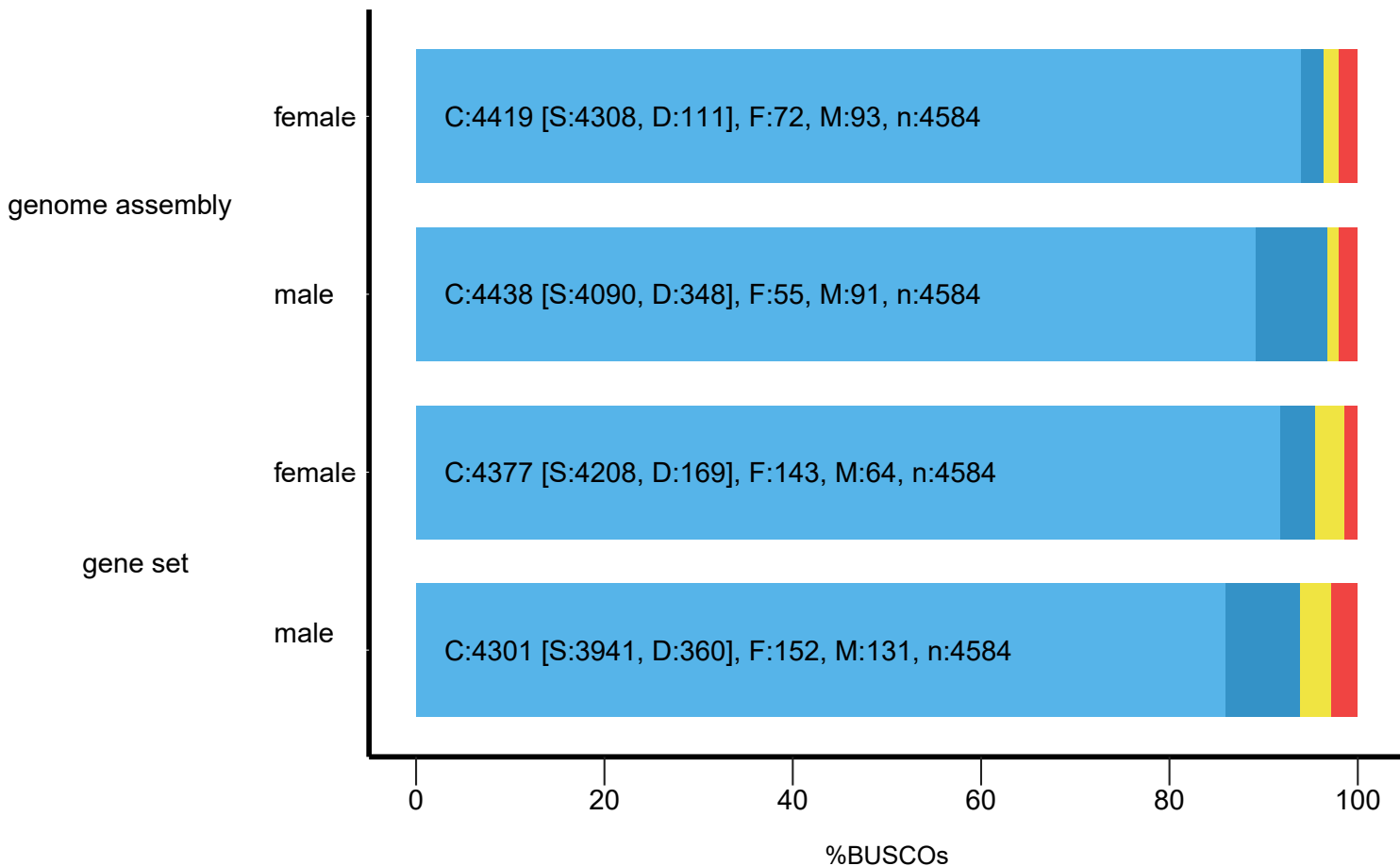

Supplement: msab056_Supplementary_Data [file msab056_supplementary_data.zip › Supplementary Fig. S2.pdf]

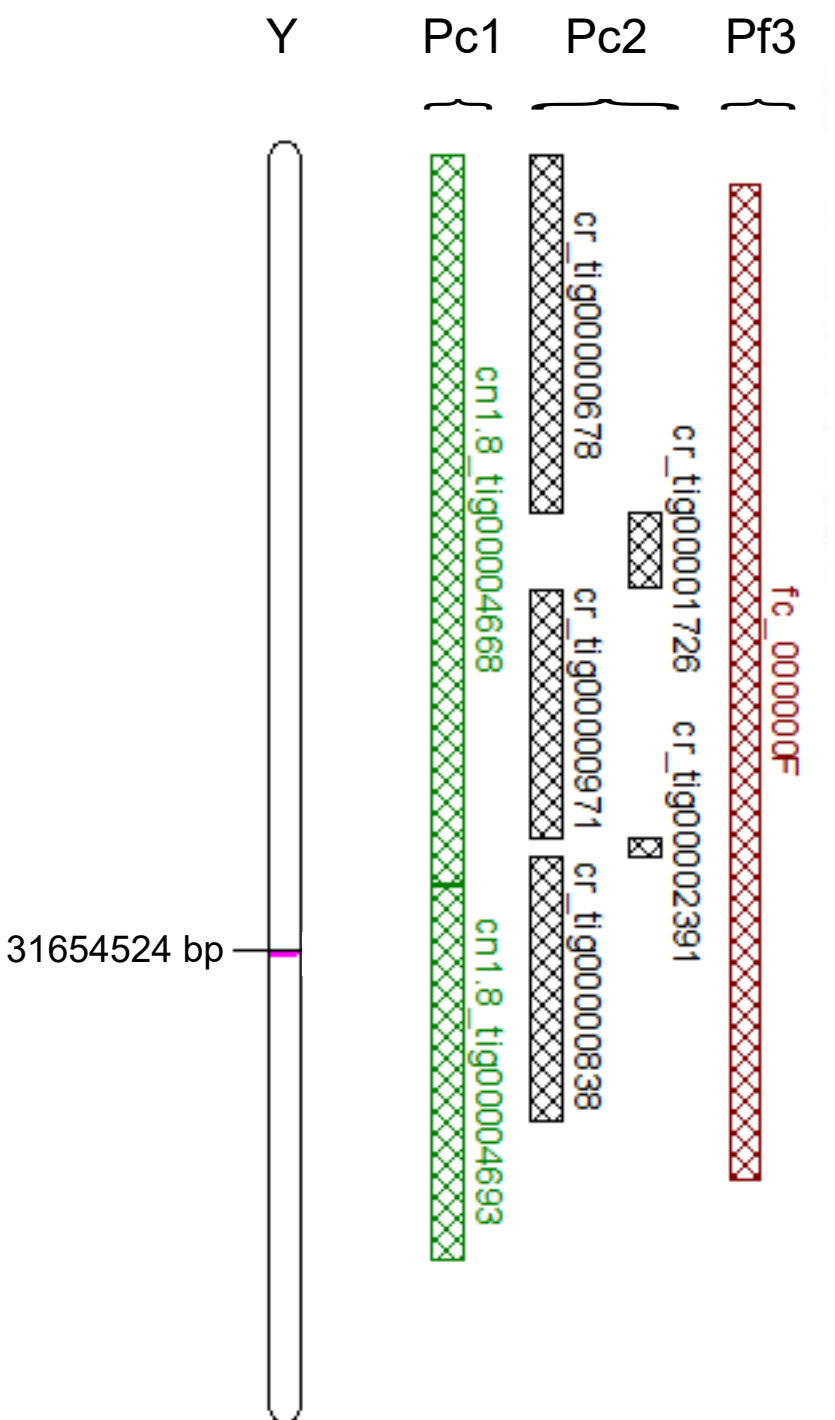

Supplement: msab056_Supplementary_Data [file msab056_supplementary_data.zip › Supplementary Fig. S3.pdf]

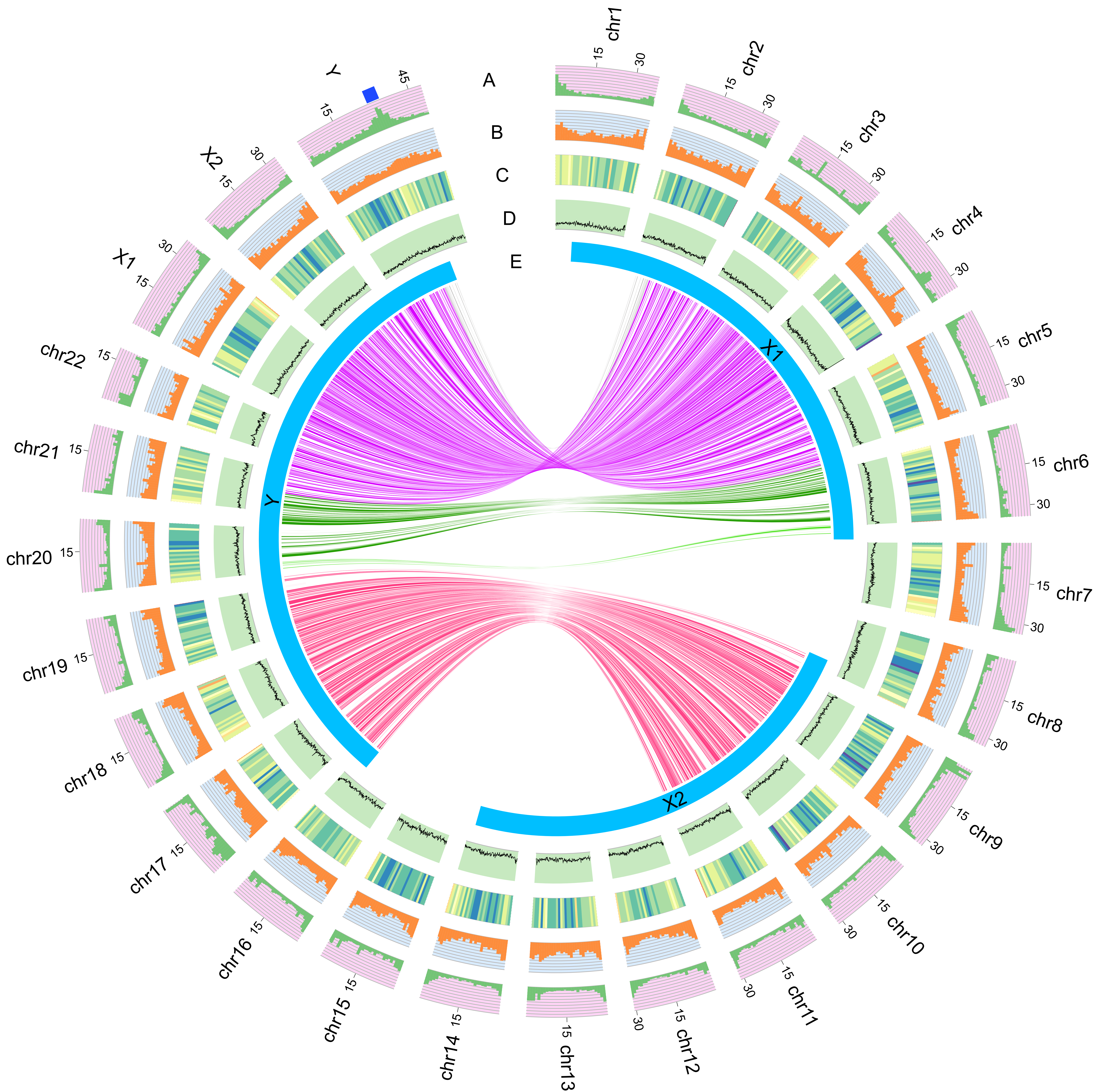

Supplement: msab056_Supplementary_Data [file msab056_supplementary_data.zip › Supplementary Fig. S4 all_chr_sex_inner_circos.pdf]

gene

PacBio

Nanopore

10X

a

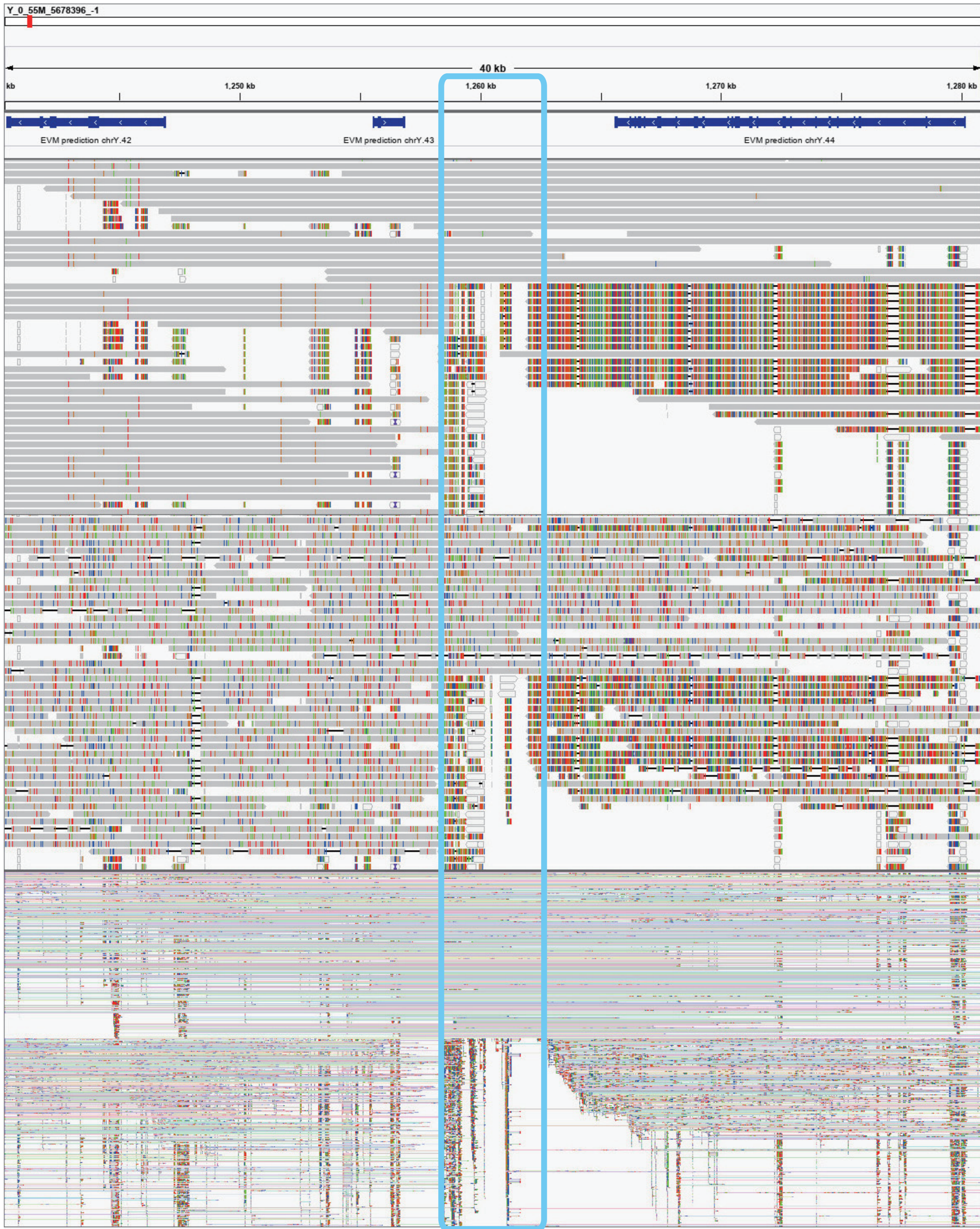

b

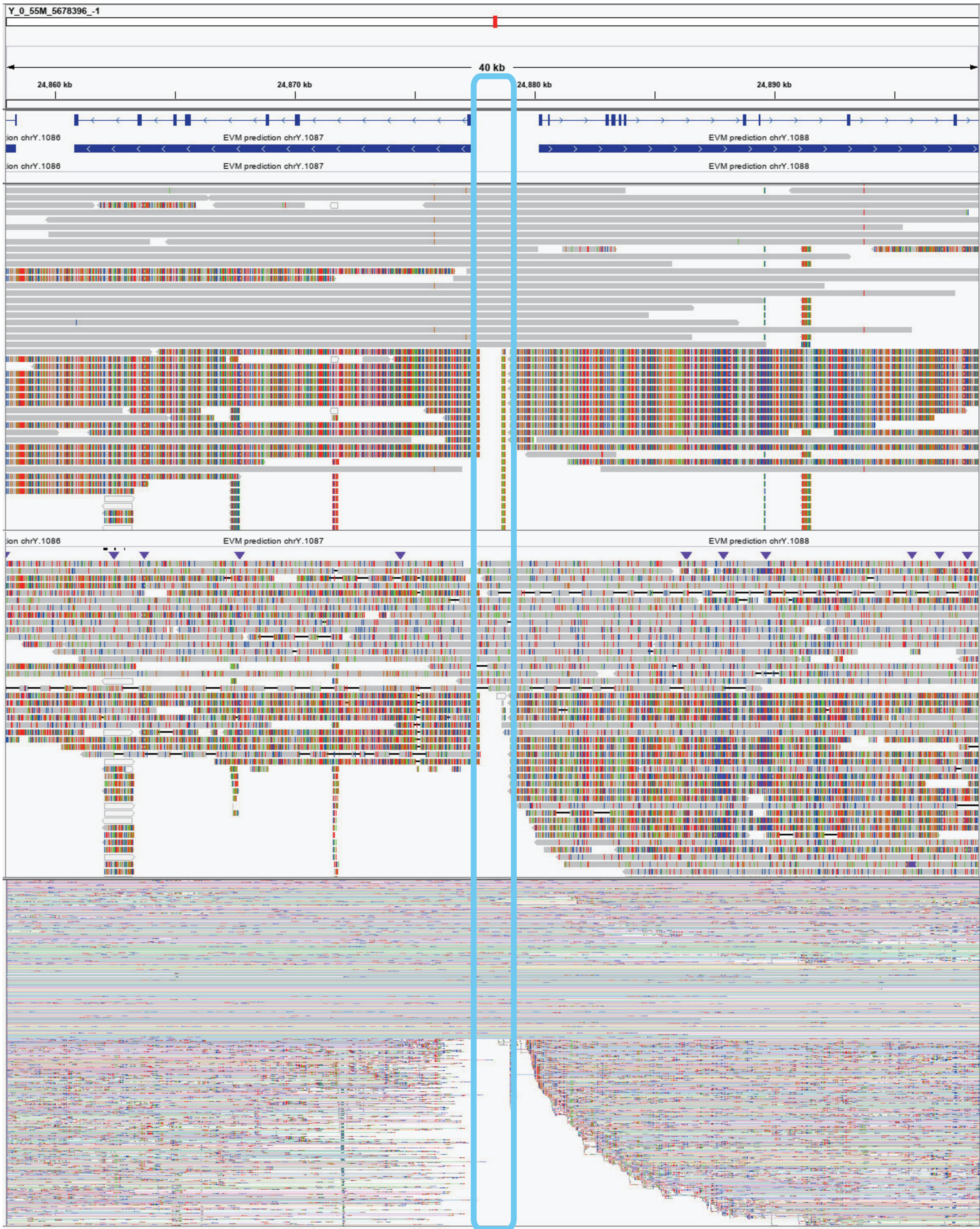

c

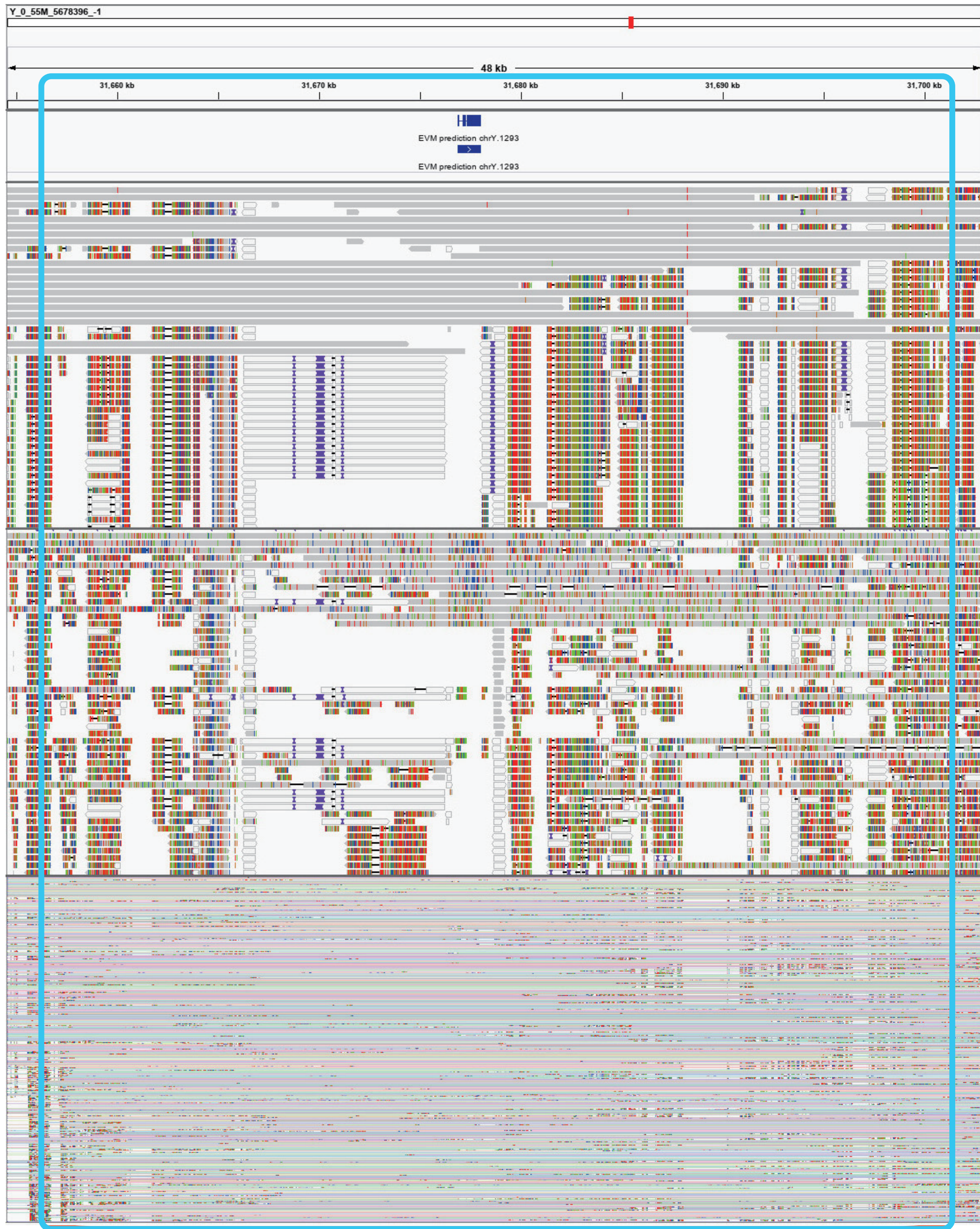

Supplement: msab056_Supplementary_Data [file msab056_supplementary_data.zip › Supplementary Fig. S5.pdf]

a

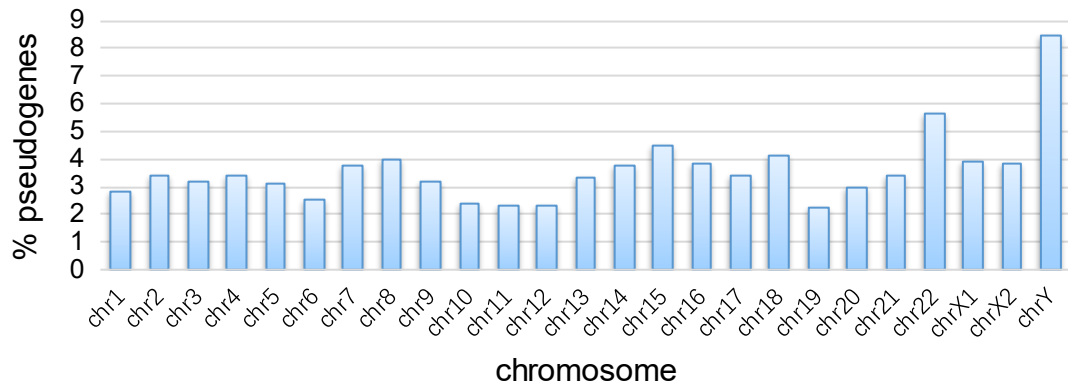

b

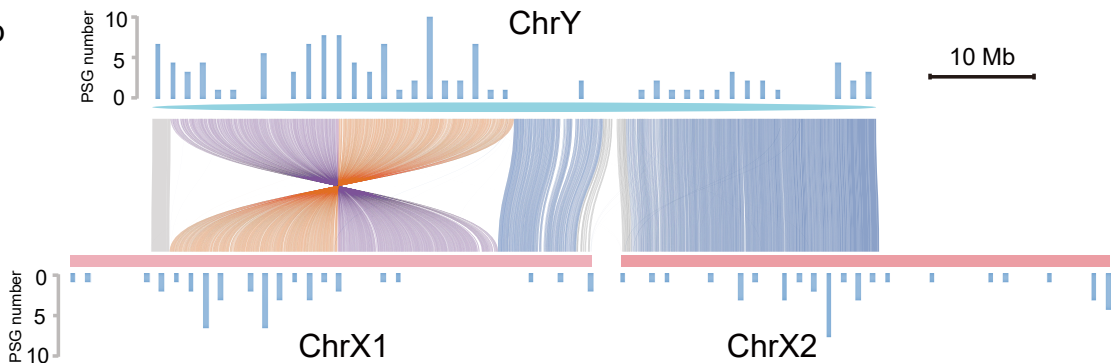

Supplement: msab056_Supplementary_Data [file msab056_supplementary_data.zip › Supplementary Fig. S6 pseudo_distribution.pdf]

## Denovo

## Homolog

Autosomes

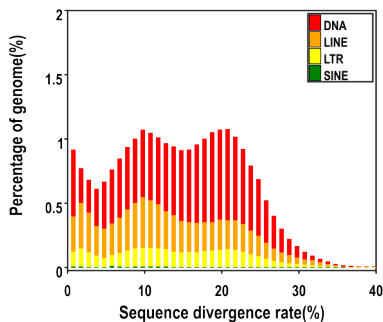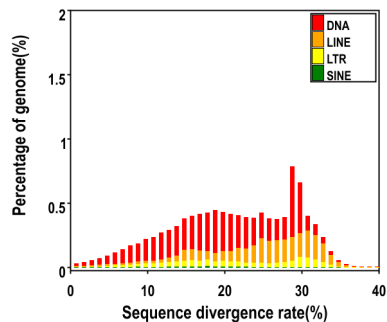

X1 and X2

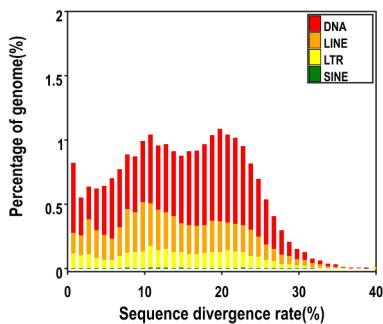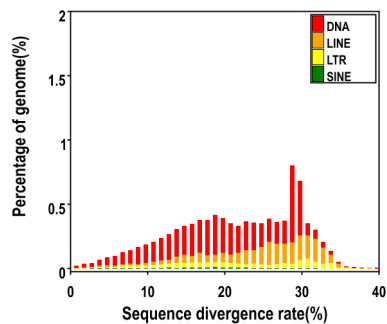

Y (non-PAR)

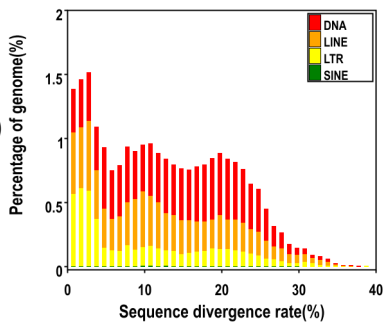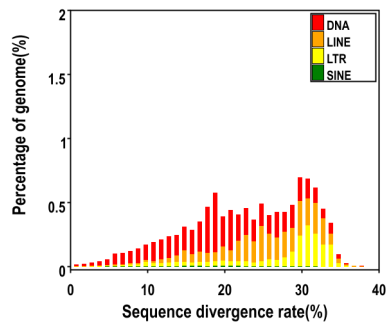

Supplement: msab056_Supplementary_Data [file msab056_supplementary_data.zip › Supplementary Fig. S7 Repeat.sequence.divergence.pdf]

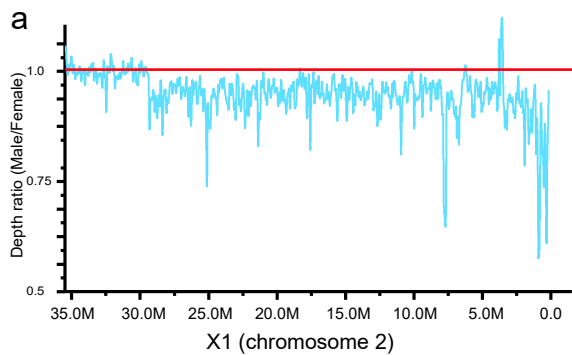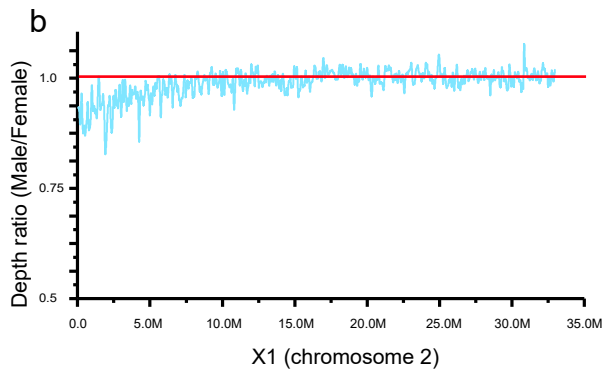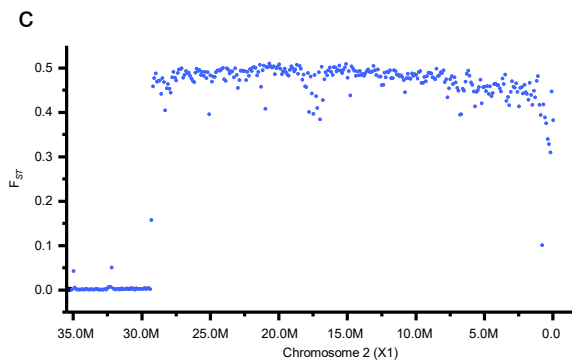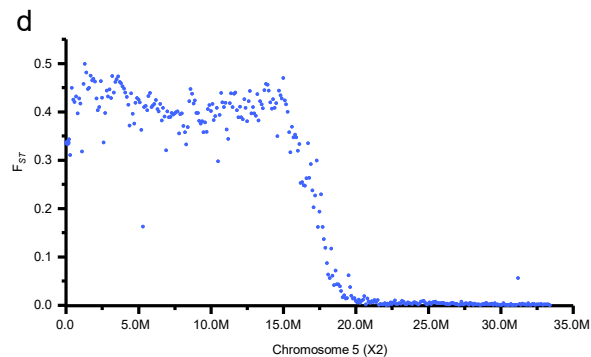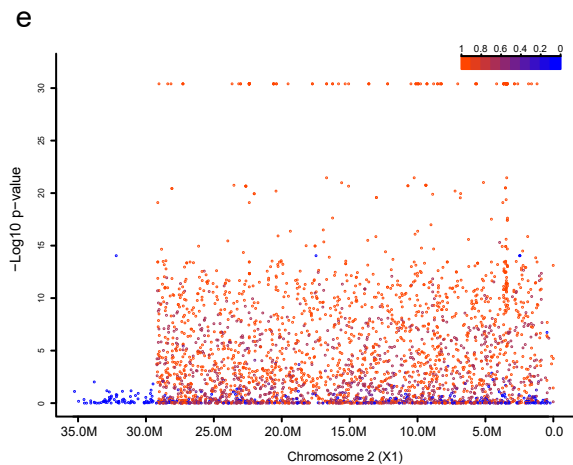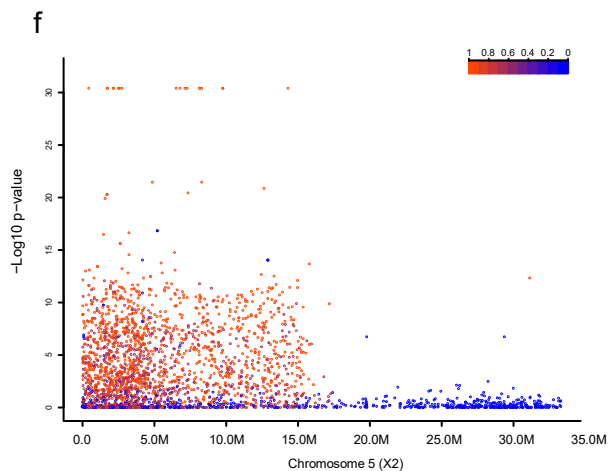

Supplement: msab056_Supplementary_Data [file msab056_supplementary_data.zip › Supplementary Fig. S8.pdf]

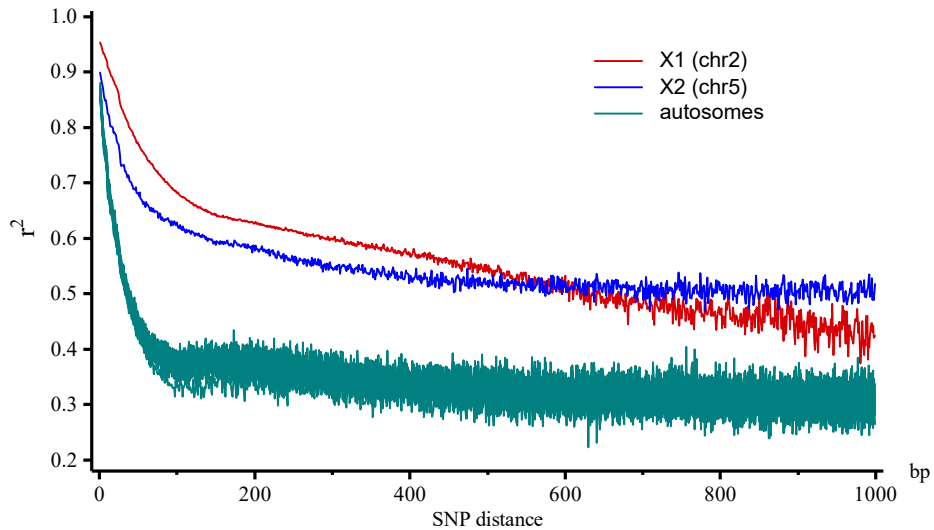

Supplement: msab056_Supplementary_Data [file msab056_supplementary_data.zip › Supplementary Fig. S9.pdf]
